# Multi-environment genome -wide association mapping of culm morphology traits in barley

**DOI:** 10.3389/fpls.2022.926277

**Published:** 2022-09-23

**Authors:** Gianluca Bretani, Salar Shaaf, Alessandro Tondelli, Luigi Cattivelli, Stefano Delbono, Robbie Waugh, William Thomas, Joanne Russell, Hazel Bull, Ernesto Igartua, Ana M. Casas, Pilar Gracia, Roberta Rossi, Alan H. Schulman, Laura Rossini

**Affiliations:** ^1^Department of Agricultural and Environmental Sciences - Production, Landscape, Agroenergy, Università degli Studi di Milano, Milan, Italy; ^2^Council for Agricultural Research and Economics, Research Centre for Genomics and Bioinformatics, Fiorenzuola d’Arda, Italy; ^3^Cell and Molecular Sciences, The James Hutton Institute, Dundee, United Kingdom; ^4^Aula Dei Experimental Station (EEAD-CSIC), Spanish Research Council, Zaragoza, Spain; ^5^Viikki Plant Sciences Centre, Natural Resources Institue (LUKE), HiLIFE Institute of Biotechnology, University of Helsinki, Helsinki, Finland

**Keywords:** culm morphology, image-analysis, lodging, multi-environment GWAS, *Hordeum vulgare*, barley

## Abstract

In cereals with hollow internodes, lodging resistance is influenced by morphological characteristics such as internode diameter and culm wall thickness. Despite their relevance, knowledge of the genetic control of these traits and their relationship with lodging is lacking in temperate cereals such as barley. To fill this gap, we developed an image analysis–based protocol to accurately phenotype culm diameters and culm wall thickness across 261 barley accessions. Analysis of culm trait data collected from field trials in seven different environments revealed high heritability values (>50%) for most traits except thickness and stiffness, as well as genotype-by-environment interactions. The collection was structured mainly according to row-type, which had a confounding effect on culm traits as evidenced by phenotypic correlations. Within both row-type subsets, outer diameter and section modulus showed significant negative correlations with lodging (<−0.52 and <−0.45, respectively), but no correlation with plant height, indicating the possibility of improving lodging resistance independent of plant height. Using 50k iSelect SNP genotyping data, we conducted multi-environment genome-wide association studies using mixed model approach across the whole panel and row-type subsets: we identified a total of 192 quantitative trait loci (QTLs) for the studied traits, including subpopulation-specific QTLs and 21 main effect loci for culm diameter and/or section modulus showing effects on lodging without impacting plant height. Providing insights into the genetic architecture of culm morphology in barley and the possible role of candidate genes involved in hormone and cell wall–related pathways, this work supports the potential of loci underpinning culm features to improve lodging resistance and increase barley yield stability under changing environments.

## Introduction

Selection of desired plant architecture traits has represented a driving force in crop domestication and breeding. In cereals, one of the most paradigmatic examples is offered by the widespread introduction of semi-dwarfing genes in the modern varieties of the Green Revolution. When high fertilizer inputs were applied, traditional varieties elongated and lodged, i.e., fell over, leading to major losses in grain yields (Islam et al., 2007; Berry, 2013; Piñera-Chavez et al., 2016). To avoid this problem, breeders developed new semi-dwarf varieties with reduced plant height and sturdy stems, improving lodging resistance and crop production (Khush, 2001; Chandler and Harding, 2013). Several semi-dwarfing genes are involved in the pathways of gibberellins (GA), brassinosteroids (BR), phytohormones that play a major role in stem elongation (Sasaki et al., 2002; Kuczyńska et al., 2013). Examples of alleles deployed in breeding include loss-of-function mutations of the rice (*Oryza sativa*) *semidwarf* (*SD1*) locus, encoding a *OsGA20ox2* involved in GA biosynthesis (Sasaki et al., 2002). In wheat (*Triticum aestivum*), mutants of *Reduced Height-1* (*Rht*) genes are responsible for the expression of mutated forms of DELLA GA signaling repressor proteins (Peng et al., 1999). In barley (*Hordeum vulgare*), *semi-dwarf 1* (*sdw1*) and *semi-brachytic 1* (*uzu1*) mutant alleles were widely used in breeding programs (Kuczyńska et al., 2013; Dockter and Hansson, 2015). Barley *Sdw1* encodes a GA 20-oxidase (like rice *SD1*), while a missense mutation in the BR receptor gene *HvBRI1* causes the *uzu* phenotype (Chono et al., 2003; Kuczynska and Wyka, 2011). Despite providing yield gains, some semi-dwarfing alleles have been associated with negative pleiotropic effects such as temperature sensitivity, late flowering, and reduced grain quality (Rajkumara, 2008; Okuno et al., 2014).

Changes in climatic conditions are predicted to increase the intensity and frequency of storms, hail, and heavy rains (Lobell et al., 2011), the major causes of lodging impacting crop productivity (Berry and Spink, 2012; Berry, 2013). In cereals such as rice, wheat, and barley, the stem or culm consists of alternating solid nodes and hollow internodes. Three different types of lodging are known: culm bending, culm breaking, and root lodging (Hirano et al., 2017a). Breaking-type lodging is more serious than bending type because bent culms are still able to transport photosynthetic assimilates, which are necessary for plant recovery and grain filling, from the leaves to the panicles. Since cereal height cannot be reduced below a certain point, the improvement of lodging resistance and, therefore, yield requires the identification and the use of other important traits (Berry et al., 2015; Dawson et al., 2015; Hirano et al., 2017a; Shah et al., 2019).

Barley is one of the most important crops worldwide. Due to its intrinsic plasticity and adaptability, barley can be cultivated in areas not suited to maize and wheat, especially where the climatic conditions are cool and/or dry. Barley varieties can be divided into two-row and six-row types. In two-row barley, the central spikelet of each triplet on the rachis is fertile, while the other two are reduced and do not develop. Mutations of the *VRS1* gene determine the fertility of these lateral spikelets to produce six-row barleys (Komatsuda et al., 2007) and have pleiotropic effects on a number of morphological traits (Liller et al., 2015).

Barley production can be lowered from 4 to 65% by lodging (Jedel and Helm, 1991; Sameri et al., 2009). While agricultural practices play an important role (Cai et al., 2019), the occurrence of culm bending/breaking lodging events is determined mainly by two factors: (1) the force exerted on the culm (e.g., wind-induced forces or panicle weight) (Pinthus, 1974) and (2) the mechanical resistance of the stem determined by composition and morphology (Samadi et al., 2019).

For example, in cereals with hollow internodes such as barley and rice, lodging resistance is influenced by morphological characteristics such as internode diameter and culm wall thickness (Samadi et al., 2019; Zhang et al., 2020). Wider culm diameter and thickness were shown to improve lodging resistance (e.g., in wheat) (Zuber et al., 1999). Berry et al. (2007) identified increased culm diameter and material strength and reduced wall width as the ideal combination of traits to make lodging-resistant wheat with minor impact on yield potential. Also, a stronger culm may help to improve yield by allowing increased nutritional inputs. Despite the relevance of these traits, knowledge of the genetic control of culm diameter and culm wall thickness is largely limited to studies in rice. A rice mutant with a larger stem diameter and thickness called *smos1* (*small organ size*) exhibits altered cell wall composition and is less prone to lodging (Hirano et al., 2014). The *SMOS1* gene encodes an APETALA2 (AP2)-type transcription factor (Aya et al., 2014; Hirano et al., 2014) that interacts with a GRAS transcription factor encoded by *SMOS2/DLT* to mediate cross-talk between auxin and BR signaling and regulate various culm morphology features (Hirano et al., 2017b). In rice cultivar Habataki, a variety with improved yield and large culms, two quantitative trait loci (QTLs) have been associated with culm architecture: *STRONG CULM1* (*SCM1*) and *SCM2*/*APO1* (*ABERRANT PANICLE ORGANIZATION1*) were, respectively, identified on chromosome 1 and chromosome 6 (Ookawa et al., 2010). Two additional *SCM* loci were identified from the high yielding and lodging resistant cultivar Chugoku 117, including *SCM3* which was shown to be allelic to the rice *TEOSINTE BRANCHED1* (*OsTB1*)/*FINE CULM1* (*FC1*) gene (Minakuchi et al., 2010; Yano et al., 2015; Cui et al., 2020). Recently, the mediator subunit gene *OsMED14_1* was uncovered as a new player in culm and lateral organ development through *NARROW LEAF1* (*NAL1*) gene regulation (Malik et al., 2020).

The lack of efficient and accurate phenotyping protocols has been a limiting factor in genetic dissection of culm architecture, for example, through exploration of wider genetic diversity in germplasm collections. In this context, different solutions emerged in recent years relying on high-throughput phenotyping methods based on the use of new image analysis tools with advanced software and special platforms (Agnew et al., 2017).

So far little is known about the genetic architecture underlying barley culm development and morphology. The aims of this work were to explore natural genetic diversity for culm architecture traits in barley; analyze their correlations with plant height, lodging, and phenology; and identify associated genomic regions and candidate genes through multi-environment genome-wide association studies (GWAS) on a collection of 261 European accessions. To these ends, we developed an image analyses–based protocol to accurately phenotype culm diameter and culm wall thickness and integrated the resulting data with genome-wide marker data from 50k SNP iSelect genotyping (Bayer et al., 2017).

## Materials and methods

### Plant materials, experimental design, and phenotyping

The germplasm collection considered in this study was composed of 165 two-row and 96 six-row barley lines, including both European cultivars and a set of Spanish landraces grown at two Northern and two Southern European sites, respectively (Supplementary Table 1). Southern sites were winter-sown and for these sites only we included 34 Spanish landraces that had a vernalization requirement. Barley lines were sown for two consecutive harvest years, 2016 and 2017, in four European research stations (Supplementary Table 2), except for the LUKE site (Finland), where data were collected only for 2017. Fields were organized in row and column designs with two complete replicates. Each plot covered on average 2 m^2^, and all the trials were rainfed – additional details about field trials and sowing densities are presented in Supplementary Table 2.

**FIGURE 1:**
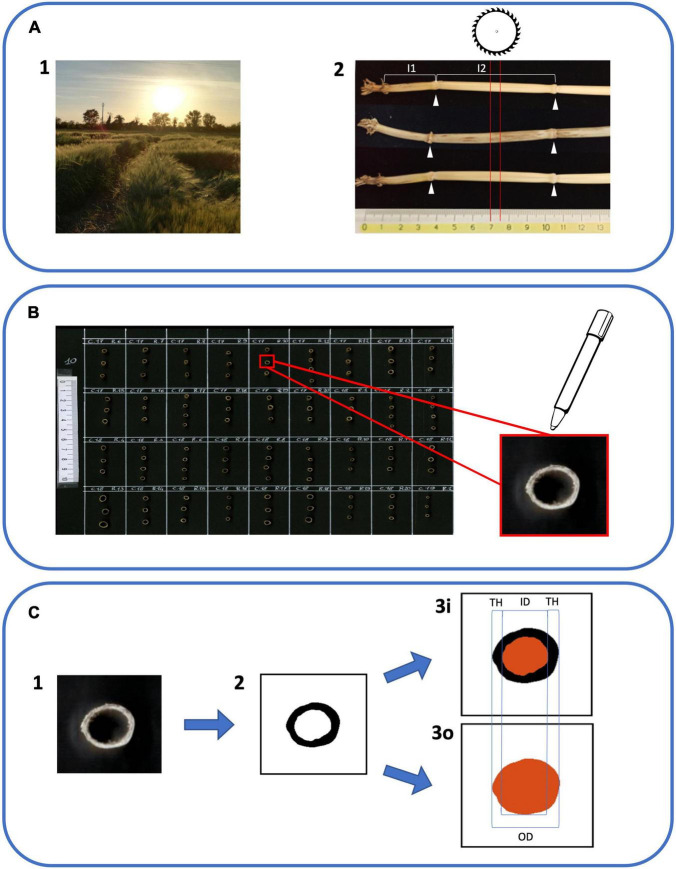
Workflow of phenotyping protocol for culm morphology traits. **(A1)** Barley specimens were gathered when plants reached Zadoks stage 90 (grain ripening). Three random plants were collected from each plot. **(A2)** Samples were cleaned and the main culm was selected for each plant. The first internode (I1) was identified as the most basal internode ≥ 1 cm. The second internode (I2) was the one immediately above (white arrowheads indicate the positions of flanking nodes). Five mm tall sections from the center of I2 (red lines) were obtained using a dedicated circular saw. **(B)** Sections were attached to black A4 cardboard with superglue and organized on the cardboard following the plot order in the field. The upper part of each section was highlighted with a bright white marker in order to enhance the contrast with the blackboard. **(C1)** Cardboards with I2 sections were scanned using a flat office scanner to obtain 300 dpi color images. **(C2)** Using the software ImageJ with a dedicated macro the I2 section images were converted to black and white images. **(C3i)** ImageJ software was able to isolate and measure the medullary cavity of the culm (in red). **(C3o)** ImageJ software was used to isolate and measure the external outline (in red). ID, inner diameter; OD, outer diameter; and TH, thickness were derived from images 3i and 3o according to formulas in Supplementary Table 3.

Zadoks scale was used throughout all trials in order to define the specific developmental stage for sampling and phenotypic measurements (Zadoks et al., 1974). Details of phenotyping methods used to measure the studied traits are described in Supplementary Table 3. Samples were collected from plot centers at Zadoks stage 90 from the second internode of the main culm, which is considered a critical area for lodging resistance (Pinthus, 1974; Berry et al., 2004). A dedicated image analysis–based protocol was developed for the measurement of culm morphological traits (Figure 1), and additional details can be found in Supplementary Method 1.

### Genome-wide single nucleotide polymorphisms genotyping and genotype imputation

The barley germplasm panel was genotyped with the 50k Illumina Infinium iSelect genotyping array (Bayer et al., 2017). Physical positions of markers were based on pseudo-molecule assembly by Monat et al. (2019) as available from the James Hutton Institute GERMINATE SNP platform (Morex v2 Assembly positions)^1^. Allele calls were made using GenomeStudio Genotyping Module v2.0.2 (Illumina, San Diego, CA, United States). After manual checking, SNP markers with more than two alleles, missing values greater than 10% and minor allele frequency (MAF) < 5% were excluded from analyses, along with unmapped single nucleotide polymorphisms (SNPs). As a result, 36020 SNP markers and 261 genotypes (165 two-row and 96 six-row barleys) remained for the analysis. Missing genotypes were imputed using Beagle v5.0 (Browning et al., 2018, Supplementary Method 2).

### Linkage disequilibrium, population structure, and kinship

In many cases, linkage disequilibrium (LD) is influenced by the presence of population structure and relatedness due to demographic and breeding history of the accessions. To take into consideration these factors, the intrachromosomal LD between two SNPs was estimated as squared allele-frequency correlations (*r*^2^) using an unbiased (due to non-independence relationships between individuals) estimation implemented in the R package called LDcorSV (Mangin et al., 2012). The markers were thinned to every third SNP, and LD between all pairs of intrachromosomal sites was estimated. Four *r*^2^ estimates were calculated: *r*^2^ based on raw genotype data, *r*^2^ with population structure represented by the principal component analysis (PCA) after scaling the PC scores across a range of zero to one (rs2, see below), *r*^2^ with relatedness (rv2; see the next section), and *r*^2^ with both population structure and relatedness (rsv2). The *r*^2^ values were plotted against the physical distance (Mb), and a non-linear LOESS curve was fitted to investigate the relationship between LD and physical distance. A square root transformation of unlinked *r*^2^ values was calculated, and the parametric 95th percentile of the distribution of transformed values was taken as a critical *r*^2^ value (Breseghello and Sorrells, 2006). The unlinked *r*^2^ refers to the *r*^2^ between the SNP loci with a physical distance greater than 50 Mb.

Population structure was estimated using PCA. Prior to PCA, the genotype marker data were filtered out by LD-pruning to generate a pruned dataset of SNPs that are in approximate linkage equilibrium, thus reducing the effect of LD on population structure. The LD-based SNP pruning was conducted with a window size of 100 kb, shifting the window by one SNP at the end of each step. Then, one SNP from a pair of SNPs was removed if their LD was greater than 0.2. Both PCA and LD pruning were conducted in the SNPRelate package in R software (Zheng et al., 2012). To investigate relatedness between individuals, a matrix of genomic relationship was calculated from marker data by the method described by Van Raden (2008), available in the R package snpReady (Granato et al., 2018).

### Statistical analysis of phenotypic data

Following a two-step approach, we initially obtained the best linear unbiased estimates (BLUEs; Supplementary Table 1) of each genotype from the analysis of individual environments. Note that in this first step, the genotype effect was treated as fixed to prevent shrinkage in estimated means. BLUEs from this first step became the phenotype input for step two for combined analysis using mixed model to estimate variance components, broad-sense heritability, and subsequent GWAS (Smith et al., 2001). The full description of analytical methods of multi-environment phenotypic data can be found in Supplementary Method 3.

### Multi-environment genome-wide association studies analysis

For GWAS, we first extended the general mixed model form of the multi-environment analysis by adding genotype principal components into the fixed part of the model. In addition, we incorporated the genomic relationships into the variance–covariance matrix of random effects to reflect the genetic relatedness between individuals in the population (Σ_*G*_⊗*K*), and allowing a diagonal residual matrix (different residual variances in each trial; *R* = ${⊕}_{i=1}^{7}R_{i}$). GWAS was performed using the method proposed by Korte et al. (2012), which can be extended to multi-environment trials to identify QTL/SNPs either with main or interaction effects. The full description of analytical methods of multi-environment GWAS can be found in Supplementary Method 4.

### Analysis of co-association network between traits

For each panel, we first organized associations from all traits into a matrix with SNPs (SNPs within the same LD region were treated as a single QTL) in rows and traits in columns, which was filled with cells for corresponding marker effects and association with the corresponding trait (QM, QF, and interaction effects), after correction for population structure and kinship. The resulting matrix was then used to provide a pairwise Pearson correlations matrix between loci. The correlation matrix was subsequently used as an input matrix for network analysis. We used undirected graph networks to visualize submodules of loci using igraph package in R to visualize proximities between loci in a network plot (Csardi and Nepusz, 2006). Nodes (SNPs) were connected by edges if they had a pairwise association above the threshold (*r* ≥ 0.9) from the similarity matrix described above.

## Results

### Diversity, population structure, and linkage disequilibrium of the barley panel

The barley panel considered in this study is a collection representing the diversity of European barley from the 20th century and was chosen based on previous geographic and genetic diversity analysis (Tondelli et al., 2013). This panel was supplemented with 57 six-row and five two-row Spanish landraces representing the ecogeographic diversity of barley cultivation in the Iberian Peninsula. Eight of the 269 genotypes did not match with their phenotypes and were discarded from the analyses, resulting in a total of 261 barley cultivars and landraces comprising 165 two-row and 96 six-row barleys being considered in this study (Supplementary Table 1). The 50k SNP iSelect genotyping of the collection yielded a set of 33342, 26262, and 27583 polymorphic markers for the whole, two-row, and six-row panel, respectively (Supplementary Table 4 and Supplementary Figure 1).

Genetic structure of the panel was investigated using PCA on a pruned subset of markers to reduce the effect of LD on population structure. PCA indicated the first two PC scores explained, respectively, 13 and 8.5% of total variation (Supplementary Figure 2A). The first PC could distinguish six-row from two-row barleys, while the second PC axis was attributed to the separation of landraces from cultivars within six-row barleys. In addition, PCA revealed a wider level of genetic variation within six-row barleys, although the proportion of two-row barleys was higher in the panel.

As a prerequisite for GWAS, LD was calculated for each chromosome using the squared correlation coefficient between marker pairs, *r*^2^, after correcting for genomic relatedness. The LD decay was visualized by plotting *r*^2^ values against the physical distance in Mb. Considerable variation was observed across the genome among the whole panel and row-type subsets, reflecting breeding history and effect of selection (Supplementary Figures 2B–D). The level of LD decay in the two-row panel at the critical *r*^2^ threshold was higher (LD = 1.4 Mb) compared to LD decay observed within the six-row panel (LD = 0.6 Mb), with slightly higher LD in the whole panel (LD = 0.8 Mb).

### Phenotypic variation, trait heritability, and correlations

The barley collection was grown under field conditions in seven environments including four locations and 2 years, 2016 and 2017 (Supplementary Table 2). Field sites were chosen to represent contrasting environments in Southern Europe (Italy, CREA; Spain, CSIC) and Northern Europe (Scotland, JHI; Finland, LUKE). Regarding culm traits, we focused on culm features reported in the literature as critical for lodging resistance in hollow cereals (Ookawa et al., 2010). Because of the great plasticity of the first internode, we decided to focus on the second basal internode as a critical point for lodging resistance and a good descriptor of culm characteristics (Pinthus, 1974; Berry et al., 2004). For all trials, outer culm diameter (OD), inner culm diameter (ID), and culm thickness (TH) were quantified using a newly developed image analysis–based protocol (Figure 1 and Supplementary Method 1). In order to investigate the correlations between culm traits and some agronomic traits, we also included heading (HD), plant height (PH), and lodging (LG) (Supplementary Table 3). We further derived section modulus (SM), the ratio between OD and TH (herewith designated as stiffness, ST) and the ratio between OD and PH (stem index, SI) as indexes reflecting the physical strength of the culm (Supplementary Table 3; Mulsanti et al., 2018; Sowadan et al., 2018). For trial CSIC16, it was not possible to collect lodging data. The best linear unbiased predictions (BLUEs) were calculated for the downstream analyses.

The single and across environment means, standard deviations (SDs), ranges, minimum, and maximum values are indicated in Supplementary Table 5. Considerable phenotypic variation was present both within and across environments. In general, higher mean values were observed for Southern environments for all traits. CSIC16 had the highest values for almost all culm traits in the whole panel, and both two-row and six-row panels. The highest values for HD were recorded in the CREA17 trial, while CREA16 had the highest mean value for PH in the whole panel and also two-row and six-row panels. Heritability values were calculated both in single and in combined environments in the whole panel and both two-row and six-row subsets (Table 1 and Supplementary Methods 3). In most environments, analysis of variance correcting for field trends i.e., the correlation between residuals from neighboring plots using the first-order autoregressive model (AR1), improved the precision compared to base model fitting. High heritability values (>50%) were obtained for most traits except for TH and ST, although these traits showed improved heritability in the combined environment analysis compared to single environment. Heritability estimates varied among environments indicating the presence of heterogeneity of genotype variance due to genotype × environment interactions. This was especially evident for TH and ST due to their relatively low heritability values.

**TABLE 1:** Estimates of broad-sense heritability for culm morphological traits in single and across environments.

Env	Factor^a^	Residual ^b^	Panel	HD	PH	OD	ID	TH	SM	ST	SI
CREA16	Gen + Rep + Col + Row	AR1(Row) : AR1(Col)	Whole panel	98.75	90.00	80.48	81.12	53.31	75.97	59.79	87.81
			2-row	96.79	82.64	65.44	69.33	**27.95**	53.13	53.36	83.82
			6-row	99.32	87.43	86.14	87.90	55.09	83.87	67.24	90.21
CREA17	Gen + Rep + Col	ID(Row) : AR1 (Col)	Whole panel	94.02	85.76	73.84	65.35	67.01	75.59	51.75	86.42
			2-row	87.47	82.84	70.22	64.19	**47.37**	67.04	56.27	82.89
			6-row	95.89	88.77	79.01	70.85	77.18	82.85	**39.37**	90.70
CSIC16	Gen + Rep + Col + Row	ID(Row) : ID (Col)	Whole panel	97.13	94.84	79.58	71.65	77.51	81.93	52.39	-
			2-row	94.39	90.15	75.29	67.85	54.87	73.45	**32.22**	-
			6-row	98.35	92.59	81.16	76.19	78.27	85.01	64.09	-
CSIC17	Gen + Rep + Col+Row	AR1(Row) : AR1(Col)	Whole panel	94.17	93.11	87.39	81.67	78.77	86.67	72.08	92.81
			2-row	86.88	85.18	79.64	78.16	**29.82**	69.25	70.15	89.71
			6-row	96.51	92.85	85.95	82.89	78.08	87.43	**48.81**	94.76
JHI16	Gen + Rep + Col + Row	AR1(Row) : AR1(Col)	Whole panel	95.62	93.85	89.54	87.51	81.98	90.00	73.07	92.15
			2-row	90.85	93.48	77.53	68.67	70.85	72.26	55.07	91.87
			6-row	87.97	90.45	88.57	89.22	76.56	89.35	74.01	91.14
JHI17	Gen + Rep + Col + Row	AR1(Row) : AR1(Col)	Whole panel	93.37	93.13	84.64	78.33	53.32	86.75	**47.19**	85.73
			2-row	83.68	92.78	69.05	63.14	**20.85**	59.37	**35.96**	85.22
			6-row	86.67	93.60	85.45	78.39	67.04	88.21	**41.48**	81.15
LUKE17	Gen + Rep + Col + Row	ID(Row) : ID (Col)	Whole panel	95.39	96.74	84.74	84.87	61.62	84.75	60.60	92.04
			2-row	91.05	93.33	57.80	57.34	**41.01**	50.03	**43.83**	91.04
			6-row	96.44	95.74	84.62	86.14	54.34	84.50	62.32	89.19
Combined Environments			Whole panel	93.10	91.42	89.21	86.98	81.52	84.77	70.49	91.91
			2-row	90.43	94.26	83.31	81.37	57.81	72.38	57.98	92.48
			6-row	89.95	81.90	90.18	88.98	68.61	85.49	75.11	91.58

Heritabilities with less than moderate values are indicated in bold. ^a^Gen, random genotype effect; Rep, Random replicate effect; Row, Random row effect; Col, random column effect; ^b^ID (Row):ID(Col), two dimensional independent error structure; ID(Row):AR1(Col), One dimensional correlated error for columns with first-order autoregressive process; AR1(Row):ID(Col), one dimensional correlated error for rows with first-order autoregressive process; AR1(Row):AR1(Col), two dimensional correlated error with first-order auto regressive process. HD, heading date; PH, plant height; OD, outer diameter; ID, inner diameter; TH, thickness; SM, section modulus; ST, stiffness; SI, stem index.

The diversity of phenotypic values according to row-type and germplasm source within the panel was visualized based on the box plots (Supplementary Figure 3). According to the box plots, diversities were highly variable across studied traits and were dependent on environment, row-type, and germplasm sources. The distribution of phenotypes was higher between four groups than within groups. In general, cultivars exhibited wider distributions for PH and culm traits; however, the diversity within landraces was comparable despite their limited sampling area. LG showed a wide range of values although the frequency of extreme values in Northern locations, especially within two-row cultivars, was highest compared to other traits. In contrast, the lowest range of phenotypic values was observed for HD both in Northern and in Southern locations.

We further compared phenotypic means according to row-type and germplasm sources as these were important factors shaping population structure within the panel (Supplementary Figure 4 and Supplementary Table 6). Results showed that two-row landraces and six-row cultivars had latest and earliest heading, respectively, in Southern trials, while two-row cultivars were latest heading in Northern trials. In these comparisons, however, it should be noted that only 6 two-row landraces were included in our collection, all from Spain, providing limited representation of this category. PH was highly variable across environments and was mainly highest for six-row landraces in Southern trials, but this was highest for mainly two-row landraces in Northern locations. LG was lowest in all environments in two-row cultivars and highest in six-rowed landraces. For culm morphology, six-row cultivars showed the highest values of OD, ID, SM, SI, and TH, whereas two-row landraces were the lowest almost in all environments. ST was however, highly variable both within and between Northern and Southern trials. Based on phenotypic values obtained from a combined analysis of environments, higher values were observed for culm morphological traits in the cultivar gene pool, especially in six-row cultivars, but two-row cultivars were on average less susceptible to lodging. Generally, landraces showed higher values for PH and HD.

Together, these analyses show that our germplasm panel harbors significant genetic variation for culm-related traits and suggests the existence of complex genotype x environment interactions. The obtained datasets provide an ideal starting point for investigating the genetic architecture of barley culm morphology under contrasting environmental conditions.

**FIGURE 2:**
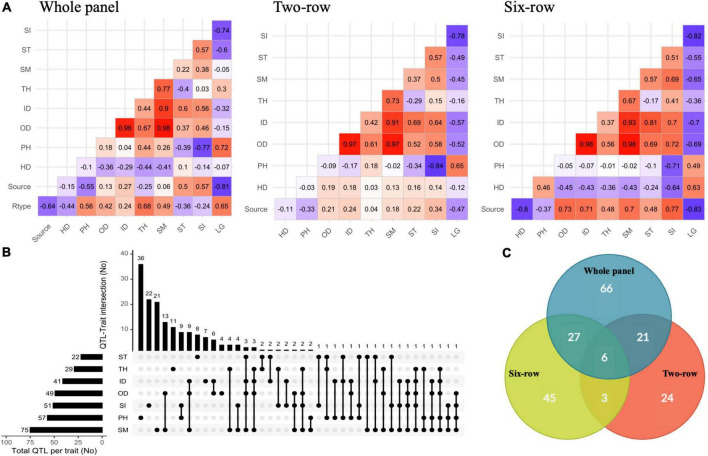
**(A)** Pairwise phenotypic correlations between traits along with row type and germplasm sources within whole panel and row type groups based on means estimated across trials. **(B)** UpSetR plot showing the overlap of the associated SNPs/loci for traits identified by GWAS. **(C)** Venn diagram showing distribution of QTLs among whole panel and row type groups. Rtype, spike row type (two-row or six-row); Source, germplasm source (cultivar or landrace); HD, heading date; PH, plant height; OD, outer diameter; ID, inner diameter; TH, thickness; SM, section modulus; ST, stiffness; SI, stem index; LG, lodging.

In order to gain insight into the relationships among different traits, pairwise correlations were calculated based on phenotype values estimated both within single and combined analyses of environments (Figure 2A and Supplementary Figures 5–7). Germplasm source and row-type were also considered, to study their relationship with the different traits. These values were also calculated within two-row and six-row panels to control for row-type. In the whole panel, row-type showed positive correlations with LG, PH, and culm morphological traits but negatively correlated with ST, SI, and HD. Germplasm source (cultivars coded as presence) had negative correlations with PH, TH, and LG and positive correlations with OD, ID, SI, and ST, meaning that cultivars were shorter and less prone to lodging with larger culm diameter compared to landraces. However, the correlation between germplasm source and HD was dependent on the region, with positive values in Northern environments and negative values in Southern sites. Results show that strong correlations were present in the whole panel between culm morphological traits. Similar results were also obtained in single environments. Except for TH, culm traits were negatively correlated with LG and HD but positively correlated with PH. As expected, LG was positively correlated with PH. Taken together, correlation analyses in the whole panel show that in our collection six-row lines tended to have wider and thicker culms and were overall more prone to lodging compared to two-row. While a confounding effect of row-type may account for the relatively weak correlations between LG and culm diameter and thickness, it should be also noted that in our germplasm collection landraces are more represented in the six-row subset compared to the two-row subset: this may be a confounding factor contributing to observed differences between the row-type subsets.

In order to explore the relationships between culm traits and lodging, excluding the effect of row-type, further analyses were conducted within row-type subsets.

In the two-row panel, correlations between culm traits were generally maintained and stronger negative correlations were observed between culm morphological traits and lodging. Some discrepancies were also observed compared to the whole panel, e.g., the negative relationship between TH and lodging in contrast to the positive correlation between these traits in the whole panel, which was possibly due to confounding effects from six-row landraces (thick culms and more prone to lodging). Furthermore, while positively correlated with lodging, PH was environment-dependent and did not show strong correlations with culm morphology, e.g., in Southern environments, the relationship was mainly weakly negative and in Northern weakly positive (Supplementary Figure 6). HD was also mainly positively correlated with culm morphology.

In the six-row panel, culm morphological traits had the strongest interrelationships. HD was also in agreement with the whole panel with stronger negative correlations with culm morphology, and in contrast to the two-row panel, it was positively correlated with lodging. PH had a negatively weak relationship with culm traits with stronger positive correlations in Northern trials and negative correlations in Southern trials (Supplementary Figure 7).

Together, these results highlight the potential of culm morphological traits as interesting targets for the improvement of lodging resistance in barley. In particular, the general lack of correlation within row-type subsets suggests that culm diameter is largely controlled by distinct genetic factors with respect to PH.

### Multi-environment genome-wide association mapping

We performed GWAS using multi-trait mixed model (MTMM) proposed for multi-trait or multi-environment association mapping to detect quantitative trait loci (QTLs) underlying culm morphological traits, incorporating kinship estimated from marker data and population structure using principal components (Korte et al., 2012). This method allows us to identify five types of marker-trait associations: markers with main effects stable across environments (QM), markers with main but also significant interaction effects (QF), marker-by-environment interaction effects (QE), marker-by-location interaction effect (QL), and marker-by-year interaction effect (QY) (see Supplementary Method 4 for more details). GWAS of multi-environment trials were performed for the whole panel and also for two-row and six-row subsets separately. The experiment-wise GWAS significance threshold was determined according to the actual number of independent SNP tests as estimated in Haploview software using the tagger function, and the *r*^2^ threshold estimated from LD decay analysis. These threshold values were found to be –log10 (*P*) ≥ 4.94, –log10 (*P*) ≥ 4.75, and –log10 (*P*) ≥ 5.02 for the whole panel, two-row, and six-row panels, respectively. However, the *p*-values with –log10 (P) ≥ 4 were also retained as suggestive QTLs.

A total of 732 marker-trait associations were detected, and the associated SNPs with -log10 (*P*) ≥ 4 in close vicinity were grouped into a single QTL based on the average LD decay, due to variable LD blocks for individual chromosomes and thus a variable decay across the chromosomes (Supplementary Figures 2B–D). This allowed us to converge marker-trait associations into 192 QTLs (93 single SNPs and 99 SNP clusters) across the whole, two-row, and six-row panels (Supplementary Table 7). From these loci, 109 were trait-specific and the remaining were co-associated with at least two traits (Figure 2B). PH with 36 QTLs and OD with four QTLs were the traits with the maximum and the minimum number of specific QTLs. Most QTLs were co-associated between culm morphological traits. Among the highest number of co-associated QTLs, 13 QTLs were common between SM and OD; 9 QTLs between PH and SI; 9 QTLs between ID, OD and SM; and 6 QTLs were commonly associated with ID and OD. In agreement with a largely independent genetic control, the lowest number of co-associated QTLs was identified between PH and culm morphological traits. In addition, 66, 24, and 45 QTLs were specific to the whole panel, two-row, and six-row panels, respectively (Figure 2C). Other QTLs were in common between at least two panels.

**FIGURE 3:**
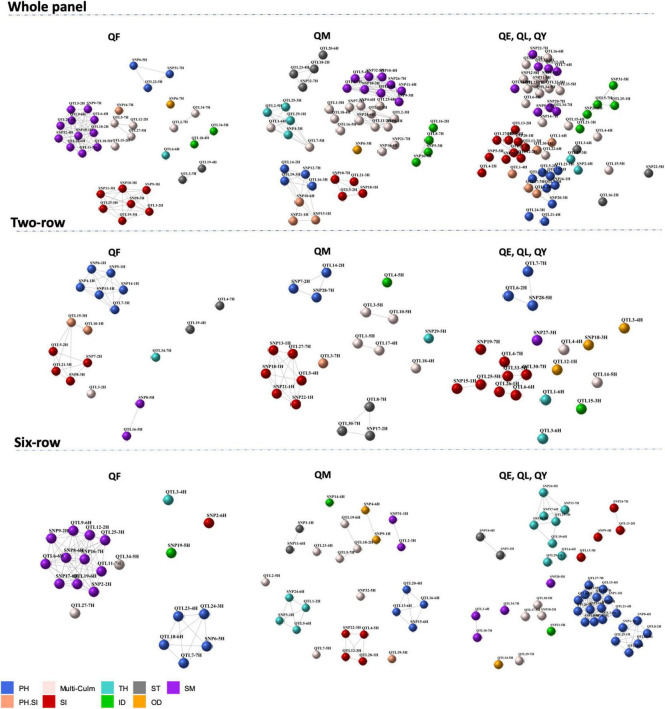
Co-association network representing co-association modules between 192 loci across whole panel and row type subsets, with color schemes according to the phenotypic traits. Each node is an SNP/QTL and a color according to its association with the corresponding trait. Strong co-associations with a correlation above threshold (*r* = 0.9) are connected by edges. PH, plant height; OD, outer diameter; ID, inner diameter; TH, thickness; SM, section modulus; ST, stiffness; SI, stem index; multi-culm stands for loci associated with multiple culm traits; PH.SI stands for loci associated with both PH and SI. QF indicates markers with main but also interaction effects; QM indicates markers with main effects and stable across environments; QE indicates marker-by-environment effect; QL indicates marker-by-location effect; QY indicates marker-by-year effect.

Co-association network analysis for the 192 QTLs revealed many co-association modules across the whole panel and the row-type sub-panels, each of which contained loci from one or more genomic regions distributed on different chromosomes (Figure 3). The co-association module is a cluster of one or more loci that are connected by edges. The edges connecting two loci have similar associations with the phenotype with a distance below the threshold. Loci in different clusters are more dissimilar than those in the same group and would not be connected by edges in a co-association module. In other words, associated nodes with edges appeared in close proximity, while weakly associated nodes appear far apart. The “Multi-Culm” group stands for multiple culm traits because these loci showed associations with multiple culm traits in the GWAS analysis. Likewise, the “PH.SI” stands for loci associated with both PH and SI in the GWAS analysis. One common feature that can be clearly derived from this visualization was that PH and SI were in closer proximity across all panels and nodes for culm morphological traits were closer together and far apart from PH. The other feature was the absence of a clear co-association module between loci associated with multiple traits, especially in the “Multi-Culm” group exhibiting higher dispersion compared to loci associated with a single trait. Furthermore, loci associated with multiple traits indicated connections with other co-association modules (e.g., with SM and TH in the whole panel; Figure 3) that might be a pleiotropic feature of these loci.

Collectively, multi-environment GWAS results identified loci controlling culm morphology independent of plant height, with some QTLs showing stable effects across environments.

### Identification of quantitative trait loci with main and full effects and putative candidate gene exploration

**FIGURE 4:**
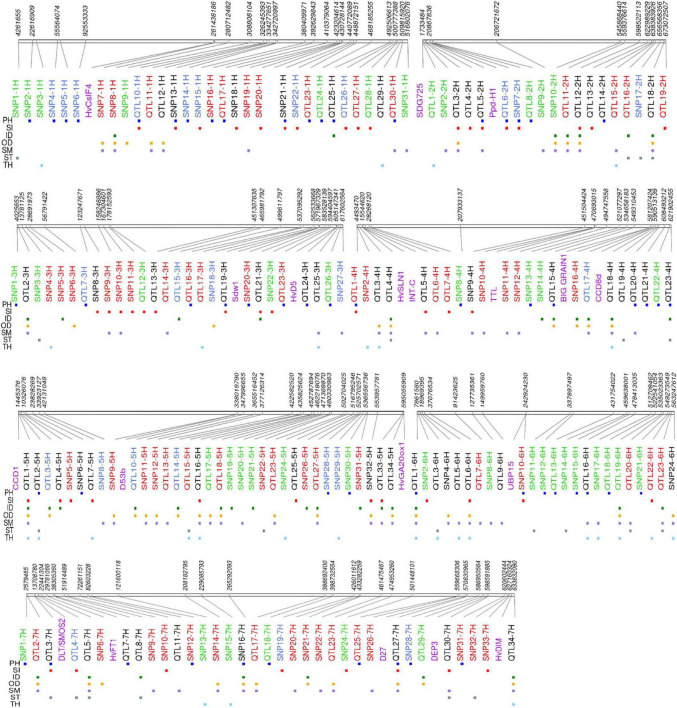
Physical map of 192 QTLs associated with culm morphological traits a cross whole panel and the row type subsets. Loci with red, blue, and green colors are unique to whole panel, two-row, and six-row subsets, respectively. Loci with black color are those detected at least in two association panel. Purple color indicates relative position of barley known genes at that particular genomic region. PH, plant height; OD, outer diameter; ID, inner diameter; TH, thickness; SM, section modulus; ST, stiffness; SI, stem index.

In Table 2, we listed the most significant QTLs associated with the studied traits with QM or QF effects and potential candidate genes. The list of all 192 QTLs with complete details can be found in Supplementary Table 7, and a synthetic view of genomic positions of QTLs along with the circular heatmap can be found in Figure 4 and Supplementary Figure 8. Promising candidate genes were selected based on literature searches, after excluding hypothetical genes and transposable elements. Marker-trait associations were listed with progressive numbering along with chromosomes: as an example of the 93 loci detected by single SNPs, SNP1-1H is the first associated locus on chromosome 1H. The 99 QTLs detected by SNP clusters are designated as QTLs, e.g., QTL10-1H.

**TABLE 2:** Summary of candidate genes underlying the most significant markers with QM and QF effect on studied traits using multi-environment GWAS.

QTL ID	Peak marker	Chr	Pos	QTL region (bp)	Panel	QTL type	Trait (–Log_10_ P)	Gene	GeneID_MOREX.V2
SNP4-1H	JHI-Hv50k-2016-19014	1H	55564074	55564074	2-row	QF	PH (5.61)	*Aspartic proteinase*	HORVU.MOREX.r2.1HG0014120
SNP5-1H	JHI-Hv50k-2016-19267	1H	59577895	59577895	2-row	QF	PH (5.61)	*Y14a*	HORVU.MOREX.r2.1HG0014550
SNP7-1H	JHI-Hv50k-2016-21372	1H	262131347	262131347	Whole-panel	QM	OD (4.71), SM (5.66)	*HvCesA4/HvClsF4*	HORVU.MOREX.r2.1HG0032090
QTL11-1H	JHI-Hv50k-2016-25138	1H	331015882	331015882–332043281	Whole-panel	QM	OD (4.71), SM (5.66)		HORVU.MOREX.r2.1HG0038730
SNP13-1H	JHI-Hv50k-2016-26359	1H	342720997	342720997	2-row	QF	PH (4.04)	*Rhmbd*	HORVU.MOREX.r2.1HG0039970
	JHI-Hv50k-2016-26359	1H	342720997		2-row	QM	SI (5.49)		
	JHI-Hv50k-2016-26359	1H	342720997		Whole-panel	QM	PH (4.6), SI (5.08)		
SNP18-1H	SCRI_RS_145336	1H	423204614	423204614	2-row	QM	SI (5.72)	*UGT707A3*	HORVU.MOREX.r2.1HG0050860
	SCRI_RS_145336	1H	423204614		Whole-panel	QM	SI (5.96)		
QTL29-1H	JHI-Hv50k-2016-52276	1H	506206295	505927936–506431159	Whole-panel	QM	TH (4.94)		Several candidate genes
QTL1-2H	12_31446	2H	1739468	1514860–1739468	6-row	QM	TH (5.01)	*SDG725*	HORVU.MOREX.r2.2HG0079410
QTL5-2H	12_31284	2H	18622308	18522424–20868342	2-row	QF	SI (5.29)	*Eligulum-a*	HORVU.MOREX.r2.2HG0086910
	JHI-Hv50k-2016-71066	2H	18623653		Whole-panel	QM	SI (4.2)		
	JHI-Hv50k-2016-71911	2H	20459215		Whole-panel	QF	SM (5.66)		
SNP7-2H	JHI-Hv50k-2016-75227	2H	28446036	28446036	2-row	QF	SI (4.75)	*BRCT*	HORVU.MOREX.r2.2HG0090080
	JHI-Hv50k-2016-75227	2H	28446036		2-row	QM	PH (4.99)		
QTL11-2H	JHI-Hv50k-2016-103173	2H	559241964	559231441–561722120	Whole-panel	QM	SM (7.23)		Several candidate genes
	JHI-Hv50k-2016-103187	2H	559376614		Whole-panel	QM	ID (5.7), OD (6.29)		
QTL12-2H	JHI-Hv50k-2016-109824	2H	598521913	597215106–598522113	6-row	QF	SM (4.27)		Several candidate genes
	JHI-Hv50k-2016-109824	2H	598521913		Whole-panel	QF	ID (4.21), OD (4.8)		
	JHI-Hv50k-2016-109823	2H	598522113		Whole-panel	QF	SM (6.16)		
QTL14-2H	JHI-Hv50k-2016-124833	2H	633916743	633545278–635684841	2-row	QM	PH (4.55)		Several candidate genes
	JHI-Hv50k-2016-124833	2H	633916743		Whole-panel	QM	PH (5.85)		
QTL15-2H	JHI-Hv50k-2016-127337	2H	638383926	638383926–638729272	Whole-panel	QF	SM (5.12), TH (4.61)		Several candidate genes
QTL18-2H	JHI-Hv50k-2016-142360	2H	665679591	665678929–669091745	6-row	QM	ID (5.09)		Several candidate genes
	JHI-Hv50k-2016-142412	2H	665806846		Whole-panel	QM	ST (4.35)		
	JHI-Hv50k-2016-142417	2H	665806970		Whole-panel	QF	SM (4.18)		
	JHI-Hv50k-2016-142979	2H	667239912		6-row	QM	SM (4.71)		
SNP9-3H	JHI-Hv50k-2016-167742	3H	158248886	158248886	Whole-panel	QM	SM (4.95)		-
QTL19-3H	JHI-Hv50k-2016-204951	3H	570921209	570921209–571967329	Whole-panel	QF	SI (4.26)	*Sdw1/HvGA20ox2*	HORVU.MOREX.r2.3HG0256590
	JHI-Hv50k-2016-204992	3H	570930550		2-row	QF	PH (5.35), SI (4.99)		
	JHI-Hv50k-2016-205354	3H	571967329		6-row	QM	PH (5.51), SI (5.74)		
	JHI-Hv50k-2016-205354	3H	571967329		Whole-panel	QM	PH (4.94)		
QTL21-3H	JHI-Hv50k-2016-206708	3H	579170749	577209764–583819782	Whole-panel	QM	SI (5.44)		Several candidate genes
	JHI-Hv50k-2016-207617	3H	583528139		2-row	QF	SI (5.47)		
QTL17-4H	JHI-Hv50k-2016-262348	4H	586245828	586245828–586286949	2-row	QM	ID (4.68), OD (4.77), SM (4.77)	*CCD8d*	HORVU.MOREX.r2.4HG0337890
QTL18-4H	JHI-Hv50k-2016-263046	4H	589723289	589666126–590513139	Whole-panel	QM	SM (4.95)		Several candidate genes
	JHI-Hv50k-2016-263069	4H	590012027		2-row	QM	SM (4.81)		-
	JHI-Hv50k-2016-263069	4H	590012027		Whole-panel	QM	OD (5.05)		
	JHI-Hv50k-2016-263064	4H	590012403		2-row	QM	TH (5.16)		
	JHI-Hv50k-2016-263064	4H	590012403		Whole-panel	QM	TH (4.73)		
	JHI-Hv50k-2016-263080	4H	590144147		2-row	QM	OD (4.92)		
	JHI-Hv50k-2016-263116	4H	590513139		Whole-panel	QF	ID (4.07)		
QTL19-4H	JHI-Hv50k-2016-263583	4H	594808440	594808131–595015320	Whole-panel	QF	ST (4.76)		Several candidate genes
	JHI-Hv50k-2016-263787	4H	594902360		2-row	QF	ST (4.86)		
QTL23-4H	JHI-Hv50k-2016-275313	4H	621344288	621344288–622035884	6-row	QM	ID (5.33), ST (4.75)		Several candidate genes
	JHI-Hv50k-2016-275693	4H	621902266		6-row	QF	PH (4.71)		
	JHI-Hv50k-2016-275696	4H	621902455		Whole-panel	QM	ST (4.16)		
QTL1-5H	JHI-Hv50k-2016-277297	5H	1444564	869533–2211050	Whole-panel	QM	OD (4.26), SM (4.2)	*CCD1*	HORVU.MOREX.r2.5HG0349440
	JHI-Hv50k-2016-277332	5H	1447495		Whole-panel	QM	TH (4.33)		
	JHI-Hv50k-2016-277338	5H	1448582		2-row	QM	SI (4.09)		
	JHI-Hv50k-2016-277724	5H	2211050		2-row	QM	ID (5.05), OD (5.6), SM (5.62)		
QTL2-5H	JHI-Hv50k-2016-278616	5H	4053376	3330549–5170277	6-row	QM	ST (5.33), TH (4.62)		Several candidate genes
	JHI-Hv50k-2016-278616	5H	4053376		Whole-panel	QM	TH (4.58)		
QTL3-5H	JHI-Hv50k-2016-279858	5H	6140678	6139160–6687421	2-row	QM	ID (4.7)		Several candidate genes
QTL4-5H	JHI-Hv50k-2016-281676	5H	10305211	10221340–10615460	6-row	QM	SI (5.24)		Several candidate genes
	JHI-Hv50k-2016-281715	5H	10326076		2-row	QM	ID (4.12)		
QTL7-5H	JHI-Hv50k-2016-287215	5H	27977719	27977719–33923320	6-row	QM	SI (4.76)		Several candidate genes
	JHI-Hv50k-2016-287531	5H	29346346		Whole-panel	QM	TH (4.12)		
	JHI-Hv50k-2016-287643	5H	29357949		Whole-panel	QM	SI (4.03)		
	JHI-Hv50k-2016-288619	5H	33923127		6-row	QM	TH (5.47)		
QTL16-5H	JHI-Hv50k-2016-309388	5H	452787194	449553280–453741857	Whole-panel	QM	TH (6.32)		Several candidate genes
	JHI-Hv50k-2016-309383	5H	452787694		2-row	QF	SM (4.3)		
	JHI-Hv50k-2016-309383	5H	452787694		Whole-panel	QM	OD (5.48), SI (4.19), SM (6.94)		
SNP19-5H	JHI-Hv50k-2016-310560	5H	467079429	467079429	6-row	QF	ID (5.79)	*ABA8ox2*	HORVU.MOREX.r2.5HG0402930
QTL27-5H	JHI-Hv50k-2016-329041	5H	525702571	525598290–525702571	Whole-panel	QF	ID (5.78), OD (5.23), SM (6.00)	*SIP1*	HORVU.MOREX.r2.5HG0420210
SNP32-5H	12_31206	5H	553957781	553957781	6-row	QM	OD (4.41), SI (7.62), SM (5.63)	*SAP6*	HORVU.MOREX.r2.5HG0430170
	12_31206	5H	553957781		Whole-panel	QM	SM (4.05)		
QTL6-6H	JHI-Hv50k-2016-383797	6H	36026739	35725637–37076534	Whole-panel	QF	TH (5.23)		-
SNP10-6H	SCRI_RS_161533	6H	242933786	242933786	Whole-panel	QM	PH (4.93), SI (4.22)	*UBP15, LG1*	HORVU.MOREX.r2.6HG0483350
QTL13-6H	JHI-Hv50k-2016-405999	6H	431754022	428846608–435119247	6-row	QM	PH (5.18)		Several candidate genes
SNP17-6H	12_30573	6H	512709462	512709462	6-row	QF	SM (5.66)	*RFP*	HORVU.MOREX.r2.6HG0509750
QTL3-7H	JHI-Hv50k-2016-449409	7H	13356822	12920299–14593868	2-row	QM	SI (5.25)	*SMOS2/DLT*	HORVU.MOREX.r2.7HG0534100
	JHI-Hv50k-2016-449409	7H	13356822		Whole-panel	QF	ST (5.87)		
	JHI-Hv50k-2016-449626	7H	13692220		2-row	QM	ST (5.95)		
QTL5-7H	JHI-Hv50k-2016-453012	7H	22070216	21643770–22444585	Whole-panel	QF	OD (4.99), SM (5.03)		Several candidate genes
	JHI-Hv50k-2016-453082	7H	22441304		6-row	QM	ID (4.7), OD (4.52), SM (4.12)		
QTL7-7H	JHI-Hv50k-2016-460460	7H	39722386	38675923–39722386	6-row	QF	PH (4.82)	*HvFT1/VRNH3*	HORVU.MOREX.r2.7HG0542540
SNP16-7H	JHI-Hv50k-2016-478948	7H	265292093	265292093	6-row	QF	SM (4.18)	*NTL*	HORVU.MOREX.r2.7HG0573190
	JHI-Hv50k-2016-478948	7H	265292093		Whole-panel	QF	OD (5.97), PH (4.46), SM (7.28)		
	JHI-Hv50k-2016-478948	7H	265292093		Whole-panel	QM	ID (6.33)		
QTL27-7H	SCRI_RS_168994	7H	570828407	570827595–572601830	6-row	QF	OD (4.74), SM (5.72)	*DWARF27*	HORVU.MOREX.r2.7HG0603370
	JHI-Hv50k-2016-493265	7H	572601830		2-row	QM	SI (4.54)		
QTL30-7H	JHI-Hv50k-2016-501203	7H	598638988	597448728–600244977	2-row	QM	ST (4.94)	*DEP3*	HORVU.MOREX.r2.7HG0610260
SNP32-7H	SCRI_RS_213791	7H	625219043	625219043	Whole-panel	QM	ST (5.05)		HORVU.MOREX.r2.7HG0620190
QTL34-7H	JHI-Hv50k-2016-516642	7H	628347284	628346780–633832080	Whole-panel	QF	ID (4.27), OD (4.07)	*HvDIM*	HORVU.MOREX.r2.7HG0622270
	JHI-Hv50k-2016-518794	7H	632545446		Whole-panel	QF	TH (5.15)		
	JHI-Hv50k-2016-519440	7H	633832080		2-row	QF	TH (5.49)		

PH, plant height; OD, outer diameter; ID, inner diameter; TH, thickness; SM, section modulus; ST, stiffness; SI, stem index. QF indicates markers with main but also significant interaction effect; QM indicates markers with main effects and stable across environments.

Out of a total of 31 QTLs on chromosome 1H, the most significant were SNP4-1H, SNP5-1H, SNP7-1H, SNP8-1H, and QTL11-1H. SNP7-1H (pos: 262.13 Mb) was associated with both OD and SM in the whole panel and located in close proximity with candidate gene *HvCesA4*/*HvClsF4*, encoding a cellulose synthase protein previously associated with culm strength in barley (Burton et al., 2010).

For chromosome 2H, 19 QTLs were detected. QTL1-2H associated with TH (six-row panel) explained a high proportion of phenotypic variance. We found that QTL1-2H (pos: 1.51–1.74 Mb) harbors the ortholog of rice *OsSDG725* encoding a histone H3K36 methyltransferase and playing an important role in rice plant growth and development (Sui et al., 2012).

For chromosome 3H, 27 QTLs were identified including QTL19-3H (pos: 570.92–571.97 Mb), which is associated with both PH and SI across all panels and is closely linked to the well-known plant height gene *Sdw1* (Dockter and Hansson, 2015) found in many elite European two-row spring barley cultivars.

On chromosome 4H, a total of 23 QTLs were identified. A particularly interesting region with QM effect was QTL17-4H (pos: 586.24–586.29 Mb) associated with ID, OD, and SM in the two-row panel and explaining at least 6% of the phenotypic variance. This QTL was found to harbor a homolog of rice *CCD8-d* (carotenoid cleavage dioxygenase). QTL18-4H was detected in both the whole panel (ID, OD, SM, TH) and two-row panel (ID, OD, SM), explaining between 2.74 and 7.1% of the variance (pos: 589.66–590.52 Mb). SNP10-4H was associated with SM and located within a pseudo-response regulator gene (470.68 Mb). Also, about 0.8 Mb from this marker we noted a homolog of *TRANSTHYRETIN-LIKE PROTEIN* (*TTL*), a gene that was previously associated with stem circumference in sorghum (Mantilla Perez et al., 2014). OD and ID were associated with SNP16-4H (481.27 Mb), 0.5 Mb from a homolog of rice *BIG GRAIN1* (Liu et al., 2015).

On chromosome 5H, 34 QTLs were detected, including three loci with promising associations. QTL1-5H was identified in two-row panel as associated with ID, OD, SM, and SI (pos: 0.87–2.21 Mb) as well as contained the rice homolog of *OsCCD1* (Ilg et al., 2009). QTL2-5H is predominantly associated in the six-row panel with PH, ST, and TH, and in the whole panel for TH (pos: 3.33–5.17 Mb) and explained more than 8% of the variance for TH and ST in the six-row panel and harbors several uncharacterized genes. SNP32-5H (pos: 553.95 Mb) was associated with OD, SI, and SM in both the six-row and the whole panel.

For chromosome 6H, in total 24 QTLs were identified, among them two SNPs with promising effect. SNP10-6H is associated with both PH and SI at position 242.933 Mb located within a gene encoding a ubiquitin carboxyl-terminal hydrolase closely related to rice *Large Grain 1* (*LG1*/*OsUBP15*), a gene involved in seed size and plant height (Shi et al., 2019). SNP17-6H (512.71 Mb) was associated with SM and TH and falls within an uncharacterized gene encoding a RING/U-box superfamily protein. A large QTL region, QTL13-6H, was associated with PH in the six-row panel (pos: 428.84–435.12 Mb) and contains several uncharacterized genes.

On chromosome 7H, a total of 34 QTLs were detected including six QTLs of special interest. QTL3-7H was associated with ST in the whole panel, and PH, SI, and ST in the two-row panel (pos: 12.92–14.59 Mb). The region contains several candidates, including a gene encoding a GRAS transcription factor orthologous to rice *DWARF AND LOW-TILLERING* (*DLT*/*SMOS2*) that can directly interact with *SMALL ORGAN SIZE1* (*SMOS1*/*RLA1*), and *RLA1* plays as an integrator with both *OsBZR1* and *DLT* to modulate their activity (Tong et al., 2009, 2012; Hirano et al., 2017b; Qiao et al., 2017).

QTL5-7H was associated with ID, OD, and SM in both whole and six-rows panels and also with ST in the six-row panel (pos: 21.64–22.45 Mb). SNP16-7H (pos: 265.29 Mb), a hotspot SNP, associated with ID, OD, and SM in the six-row and whole panels. Another noteworthy QTL was QTL27-7H, associated with PH, SI in the whole panel; OD, PH, and SM in the six-row panel; and SI in the two-row panel (pos: 570.827–572.61 Mb). The region contains *HvD27*, the barley ortholog of rice strigolactone biosynthesis gene *DWARF27* encoding beta-carotene isomerase (Lin et al., 2009). QTL30-7H (pos: 597.44–600.25 Mb) was associated with SI, SM, and TH and contains several genes including a patatin encoding protein gene highly related to *DEP3*, a rice gene previously shown to affect culm morphology and anatomy as well as panicle architecture (Qiao et al., 2011). Finally, QTL34-7H (pos: 628.34–633.84 Mb) was associated with TH in the two-row panel and with ID, OD, and TH in the whole panel. This locus had also a QL effect with SM both in the six-row and whole panel and contains *HvDIM* encoding Delta(24)-sterol reductase previously shown to act in the brassinosteroid pathway in barley (Dockter et al., 2014).

### Identification of quantitative trait loci with interaction effects

Besides the abovementioned QTLs with main and full effects, multi-environment GWAS uncovered highly significant QTLs with interaction effects. QTL26-1H (pos: 495.79–497.02 Mb) was associated with SI in the two-row panel. QTL6-2H (pos: 22.37–23.99 Mb), associated with SI and PH (whole, two-row, and six-row panels), spans the well-known barley *PPD-H1* gene (Supplementary Table 7), involved in photoperiod responsive flowering (Turner et al., 2005). The genomic region of QTL15-3H (pos: 499.61–499.87 Mb), associated with ID in two-row subset, hosted uncharacterized genes. QTL34-5H (pos: 594.17–596.71 Mb) was associated with ID, OD, and SM in the whole and six-row panels. This QTL showed QE and QF effects in the whole panel and six-row panel, respectively, and contains a barley Gibberellin 20 oxidase, *HvGA20ox1*, which has recently been associated with straw breaking and flowering time in barley (Göransson et al., 2019; He et al., 2019). QTL7-7H for PH was found across all panels and located in close proximity to the barley *HvFT1*/*VRNH3* gene. It showed QL effect in the whole and two-row panels and QF effect in the six-row panel. In barley, *HvFT1* expression requires the active version of *PPD-H1* to promote flowering under long-day conditions (Hemming et al., 2008). Currently, there is no report on its effect on plant height.

### Allelic comparison of single nucleotide polymorphisms/quantitative trait loci with QM/QF effects for lodging and plant height

In order to appraise the effects of the QTLs on lodging susceptibility, we focused on QTLs with QM and QF effects (Supplementary Figures 9, 10Supplementary Table 7). Allelic comparisons for these loci indicated that depending on the trait and subpopulation their effect was highly variable. As expected, QTLs for PH and SI showed significant differences for both PH and LG. With respect to culm morphology QTLs, the effects on PH and LG were variable ranging from no difference to significant differences, including some QTLs that significantly affected both LG and PH. However, most QTLs associated with culm morphology had no effects on PH in the whole panel but showed significant effects on LG. Such types of QTLs were also detected in both six-row and two-row panels. For example, the QTLs associated with ID, OD, and/or SM - SNP7-1H, SNP8-1H, QTL11-1H, QTL11-2H, QTL2-3H, SNP5-3H, SNP10-4H, SNP11-4H, SNP16-4H, QTL18-4H, QTL16-5H, QTL5-6H, QTL23-6H, QTL2-7H, SNP21-7H, SNP26-7H - affected lodging without any effect on PH in the whole panel. In two-row, some examples are QTL17-4H, QTL18-4H, and QTL10-5H. Finally, for six-row panel, SNP9-1H, SNP14-4H, and SNP32-5H are QTLs affecting lodging without any effect on PH. Considering loci with main effects (Supplementary Figure 9), out of 21 loci associated with OD, 11 had a significant impact on LG without any effect on PH (8 in the whole panel, 1 and 2 in the six-row and two-row, respectively), and out of 25 loci detected for SM, 16 significantly affected LG without impacting PH (14 in the whole panel, 2 in two-row): nine of these QTLs were shared between OD and SM (SNP7-1H, SNP8-1H, QTL11-1H, QTL11-2H, QTL2-3H, QTL17-4H, QTL18-4H, QTL16-5H, SNP32-5H). Interestingly, QTL18-4H was detected in both the whole panel and the two-row panel also for TH, indicating this locus as an interesting target for manipulation of culm morphology and lodging resistance. However, fewer loci associated with TH and ST had effects on LG. We thus focused on OD, ID, and SM for more detailed analyses of nine SNPs associated with these traits in the whole panel: SNP7-1H, SNP8-1H, SPN5-3H, SNP10-4H, SNP11-4H, SNP16-4H, SNP32-5H, SNP21-7H, and SNP26-7H. In all cases, alleles increasing culm diameter (OD, ID) and/or SM had negative effects on lodging, without affecting PH (Table 3).

**TABLE 3:** Details of subset of SNPs with main effects and associated with culm traits with negative effects on lodging without impacting on plant height.

SNP	Panel	Trait	Peak marker	MAF	A1	A2	chr	position	–Log10 (*P*-value)	β	α _1_	α _2_	PVE (%)
SNP7-1H	Whole panel	OD	JHI-Hv50k-2016-21372	0.13	A	C	1H	262131347	4.71	0.472	–0.012	–0.001	2.46
		SM		0.13	A	C	1H	262131347	5.66	0.533	–0.024	0.008	3.41
SNP8-1H	Whole panel	ID	JHI-Hv50k-2016-22255	0.14	C	A	1H	280712482	4.15	0.481	–0.008	–0.012	2.91
		OD		0.14	C	A	1H	280712482	4.26	0.502	–0.01	0.007	2.98
		SM		0.14	C	A	1H	280712482	4.15	0.501	–0.019	0.022	3.24
SNP5-3H	Whole panel	ID	JHI-Hv50k-2016-162361	0.21	A	G	3H	28691973	4.06	–0.255	0.003	0.003	1.53
SNP10-4H	Whole panel	SM	JHI-Hv50k-2016-246906	0.09	C	T	4H	470693015	4.21	0.554	–0.035	0.02	1.72
SNP11-4H	Whole panel	SM	JHI-Hv50k-2016-247273	0.09	G	T	4H	474202180	4.21	0.554	–0.035	0.02	1.72
SNP16-4H	Whole panel	ID	JHI-Hv50k-2016-261211	0.15	T	C	4H	581266705	4.38	0.307	–0.007	0.003	1.23
		OD		0.15	T	C	4H	581266705	4.30	0.312	0.001	0.002	1.21
SNP32-5H	Whole panel	SM	12_31206	0.27	C	G	5H	553957781	4.05	0.185	0	–0.005	1.06
	Six-row	OD		0.24	C	G	5H	553957781	4.41	0.345	–0.005	0.002	3.51
	Six-row	SI		0.24	C	G	5H	553957781	7.62	0.522	0.017	–0.042	6.62
	Six-row	SM		0.24	C	G	5H	553957781	5.63	0.358	0	–0.006	4.85
SNP21-7H	Whole panel	ID	JHI-Hv50k-2016-486762	0.19	C	G	7H	434555860	4.26	–0.482	–0.011	–0.005	4.93
		OD		0.19	C	G	7H	434555860	4.05	–0.481	–0.004	–0.02	4.48
SNP26-7H	Whole panel	SM	JHI-Hv50k-2016-492337	0.24	C	T	7H	562028351	4.23	–0.511	0.017	–0.034	7.02

A1, A2, and MAF indicate major allele, minor allele, and minor allele frequency, respectively. The allele associated with decreased lodging is underlined. PVE (%) is the percentage of phenotype variance explained by SNP. β is the SNP main effect, α_1_ is the SNP -by-location effect, and α_2_ is the SNP -by-year effect derived from GWAS model. OD, outer diameter; ID, inner diameter; SM, section modulus; SI, stem index.

In conclusion, the results from these analyses support the usefulness of SM and culm diameter as parameters for selecting alleles to improve lodging resistance and provide chromosomal positions and markers associated to promising loci.

## Discussion

In this study, we investigated natural genetic variation for morphological characteristics of the barley culm and their relationships with lodging and agronomic traits. To date, no genetic studies have used image processing-based phenotyping to investigate the genetic architecture of culm morphology in barley. For this reason, we developed a robust method to extract quantitative measurements of culm diameter and thickness from images of culm sections, showing that significant phenotypic variation exists within our barley germplasm panel with a major contribution of genetic variation to these traits as supported by medium–high heritability values.

Using PCA, we showed that row-type and germplasm sources are the major factors driving the population structure of the panel. In addition, no evidence of strong admixture between row-type groups was observed in PCA. This is consistent with previous studies suggesting that breeders largely focused within the six-row and two-row gene pools in developing new varieties, therefore, limiting the exchange of genetic variation between these major row-types, despite some cases of targeted introgression (Hernandez et al., 2020). Increasing seed number per spike was probably the reason for the human selection of recessive allele at *VRS1* into the barley gene pool during domestication (Komatsuda et al., 2007). On the other hand, barleys most commonly grown in Europe are two-row cultivars, which are preferred for malting because of uniformity in seed size: this resulted in limited genetic diversity compared to the six-row cultivars. This variation in seed size is due mainly to the allelic variation at the *INT-C*/*VRS5* gene between row-types (Ramsay et al., 2011). Row-type genes have pleiotropic effects on other traits, as well-known for tillering (Liller et al., 2015). In our study, row-type subsets exhibited clear differences also for some culm morphological traits, e.g., six-row barleys showed higher mean values of TH and SM compared to two-row barleys. Relationships between row-type and the studied traits are also evident from positive correlations with PH, OD, ID, SM, TH, and LG and negative correlations with HD, ST, and SI.

Correlation results showed that although plant height is important for lodging, culm characteristics also play an important role in lodging resistance. We observed strong positive correlations among culm traits, as well as negative correlations between culm traits and lodging across the whole and row-type panels, in line with a recent study focusing on peduncle morphology in a barley Nested Association Mapping population (Zahn et al., 2021). On the other hand, culm morphological traits showed weak (two-row) and even no (six-row panel) correlations with plant height. This suggests opportunities for genetic improvement of lodging resistance through manipulation of culm morphology independent of plant height. Generally, relationships among traits were similar across row-type subpopulations, sometimes with different magnitudes: for example, correlation between LG and OD was −0.52 and −0.69 in two- and six-row subpanels, respectively. Interesting correlations specific to the six-row subset were detected between LG, PH, and HD with six-row landraces being late heading, taller, and more prone to lodging compared to six-row cultivars: these landraces also had lower values of OD, ID, and SM, therefore, combining different unfavorable traits for lodging susceptibility. It should be noted that these contrasting patterns may be due to the different origins of the accessions: the six-row cultivars were mainly early flowering lines of Scandinavian origin, while the six-row landraces were of Mediterranean origin. Based on these observations, it would be interesting to further explore the genetic relationships among heading, plant height, and culm morphological traits in a wider sample of six-row barleys.

Based on these results, we analyzed phenotypic variation and run mixed model GWAS in the whole panel, as well as row-type subgroups independently in order to: (i) minimize the confounding effects of row-type on association analyses; (ii) understand whether distinct loci are segregating in row-type subpopulations and thus different regulatory networks are involved in genetic control of the studied traits. The use of mixed model in GWAS is a well-established approach to efficiently reduce false positive associations for most traits, but it may also mask true signals that are correlated with population structure. As a result, loci that distinguish barley subpopulations are often difficult to detect using mixed model. To circumvent this problem, many association mapping studies have analyzed each subpopulation separately and successfully identified loci specific to each subpopulation. In our study, 120 marker-trait associations were detected in the whole panel, including 21 and 27 that were shared with the two-row and six-row panels, respectively. Six associations were detected across all three panels. In addition, we uncovered 24 and 45 QTLs specific for two- and six-row panels, supporting the relevance of running GWAS on row-type subsets. We also noticed that for some QTLs detected across both row-types, allele frequencies and peak markers differed between the row-type subsets, resulting in opposite effects of minor alleles on the same trait. Taking as an example the PH locus QTL19-3H closely linked to the well-characterized *Sdw1* gene, the peak marker in the six-row panel was JHI-Hv50k-2016-205354 with the minor allele showing a negative effect on PH and positive effect on SI, in contrast to the effect of JHI-Hv50k-2016-204992, the peak marker in the two-row panel. Likewise, QTL6-2H containing *PPD-H1* had negative effects on PH in the six-row panel while the effect in two-row was the opposite. This indicates that causative variants in these major genes have different frequencies and are associated with different markers in row-type subsets. Taken together, comparative analysis of results from the whole panel and row-type subsets indicates the need to duly account for population structure in dissecting culm morphological traits and carefully analyze the effects of potentially interesting markers for breeding in relation to row-type population. This is also relevant when considering crosses between row types in the context of plant breeding.

While in our GWAS analysis we observed numerous trait-specific QTLs, we also observed QTLs that were associated with multiple traits. In addition, in the same QTL region, the peak marker was sometimes different depending on the panel. For example, QTL34-7H was associated with OD, ID, SM, and TH in the whole panel, with SM in the six-row panel, and with TH in two-row panel (QF effect, Supplementary Figure 10). The peak markers for TH were also different between the whole panel and the two-row panel, while the peak marker for SM was common between six-row and the whole panel. This QTL harbors the *HvDIM* gene encoding the barley Δ5-sterol-Δ24-reductase, an enzyme involved in the brassinosteroid biosynthetic pathway (Dockter et al., 2014). A link between brassinosteroids and culm thickness is supported by studies of the rice *SMOS1* and *SMOS2* genes, encoding transcription factors of the AP2 and a GRAS family, respectively, that interact to integrate auxin and brassinosteroid signaling: *smos1* and *smos2* single mutants, as well as *smos1-smos2* double mutants, show increased culm thickness (Hirano et al., 2017b). Classical semi-dwarf barley mutants *brh.af*, *brh14.q*, *brh16.v*, *ert-u.56*, *ert-zd*, and *ari.o* were shown to harbor mutations in the *HvDIM* gene (Dockter et al., 2014): these mutants have reduced plant height and are more resistant to lodging compared to respective wild type (Dahleen et al., 2005), but their culm morphological traits have not been described. In our work, a marker within this region showed a weak association with PH (JHI-Hv50k-2016-516979, *p* = 0.003), suggesting *HvDIM* as a possible candidate for QTL34-7H. However, this genomic region harbours other potential candidate genes, that have been reported as members of glycosyl transferase (GT) gene family, such as cellulose synthase genes of the GT2 family that influences culm cellulose content (Houston et al., 2015). Given the significance of associations between this genomic region and multiple culm morphology traits, it would be interesting to further dissect this QTL to discriminate whether such effects are the result of pleiotropy or closely linked genes (local LD) and identify the underlying gene(s)/alleles combining association mapping and biparental fine mapping.

Taking advantage of data from seven different environments, multi-environment GWAS (Korte et al., 2012) enabled us to disentangle QTLs with main effects stable across environments (QM) from QTLs with environment-dependent effects (location and/or year). An example of a QTL with significant interaction with location is QTL6-2H, which was detected for PH across all panels. This genomic region contains the well-known *PPD-H1* gene (Turner et al., 2005), a major regulator of barley flowering in response to photoperiod, that was shown to have pleiotropic effects on several agronomic traits including yield, leaf size, and plant height (Karsai et al., 1999; Digel et al., 2016). With respect to lodging, alleles with stable phenotypic effects across environments are preferable for breeding under changing climatic conditions. For this reason, we decided to focus our attention on culm morphology QTLs with main effects, showing a significant negative impact on lodging without affecting PH: for nine SNPs detected in the whole panel, alleles increasing culm diameter and/or SM consistently reduced lodging (SNP7-1H, SNP8-1H, SNP5-3H, SNP10-4H, SNP11-4H, SNP16-4H, SNP32-5H, SNP21-7H, and SNP26-7H). We scanned regions adjacent to these SNPs ± 0.8 Mb (i.e., the genome-wide LD decay estimated for the whole panel) in order to search for potential candidate genes. For example, cellulose synthase gene *HvCslF4* (1H, 261.4 Mb) is located near SNP7-1H (262.1 Mb): a retroelement insertion within this gene was previously associated with the *fragile stem2* (*fs2*) mutant phenotype in barley, suggesting a link between stem strength and genes involved in cellulose content (Burton et al., 2010). Since we analyzed culm morphology traits in straw culm sections, cell wall composition and cellulose content are likely to impact the morphological features considered in our work. Another example is SNP32-5H (5H, 553.9 Mb): the adjacent region hosts several possible candidate genes, including *HvMND1* (552.9 Mb), which encodes an *N*-acetyl-transferase-like protein recently shown to regulate barley plastochron and plant architecture (Walla et al., 2020).

Beside these SNPs, additional QTLs were identified as associated with culm features and having an impact on lodging, independent of PH. Among them, QTL17-4H had the main effects on ID, OD, and SM and contained a carotenoid cleavage dioxygenase 8 (*CCD8*) gene located in close proximity to the peak marker. A recent phylogenetic study showed that rice has four *CCD8* genes (*CCD8-a*, *-b*, *-c*, and *-d*), while *Arabidopsis* has only one: both *Arabidopsis CCD8* and rice *CCD8-b* are involved in the biosynthesis of strigolactones, phytohormones that control lateral shoot growth, and affect stem thickness at least in some species (reviewed in Chesterfield et al., 2020). The barley ortholog of *OsCCD8-b* is located on chromosome 3H, while the *CCD8* gene associated with QTL17-4H is more closely related to *OsCCD8d*, whose function has not been characterized yet (Priya and Siva, 2014). An alternative candidate gene for this QTL may be *MDP1*, encoding a MADS box transcription factor implicated in brassinosteroid signaling (Duan et al., 2006).

In conclusion, we established a new *ad hoc* phenotyping protocol to obtain accurate measurements for internode diameters and thickness and derive section modulus and other parameters known to impact lodging resistance in cereals. Application of this method to a diverse set of field-grown barley accessions under seven different environments in Southern and Northern Europe provided a unique platform to dissect genotype x environment interactions and identify stable QTLs across environments. These results support the robustness of our phenotyping protocol that may now be applied to other major crops with similar stem morphology, such as wheat and rice.

While validation of potential candidate genes will require more detailed analyses, this work represents the first comprehensive analysis of the genetic architecture of culm morphology in a barley germplasm collection and its relevance for lodging. Identification of 21 main effect loci for culm diameter and/or section modulus with significant effects on lodging, independent of plant height, may open new avenues to improve lodging resistance and increase barley yield stability.

## Data Availability Statement

The genotyping dataset used for GWAS is provided in Supplementary Table 8 and the same genotyping data in VCF (Variant Call Format) format containing SNP calls are also provided as a Supplementary material.

## Author contributions

GB developed the stem phenotyping protocols, collected the data for culm morphology traits, and contributed to writing of the manuscript. SS conducted the statistical analyses and drafted the manuscript. AT, HB, and WT multiplied and dispatched the seed for trials. AS, AT, WT, AC, and EI generated the standard scoring protocol. WT designed the field trials and matched the genotypic data with the phenotypic. AT, LC, WT, HB, JR, PG, and AS hosted field trials. SD, HB, PG, and EI collected the samples and agronomic data. RW, JR, AC, and EI provided genotypic data. RR helped in candidate gene searches. LR conceived and coordinated the study, contributed to writing of the manuscript and agrees to serve as the author for contact and responsible for communication. All authors revised the manuscript and approved its final version.

## Footnotes

1
https://ics.hutton.ac.uk/50k/
