# Supplementary material for: Multi-environment genome -wide association mapping of culm morphology traits in barley
Source: Front Plant Sci. 2022 Sep 23;13:926277. doi: 10.3389/fpls.2022.926277 (PMC9539552; doi:10.3389/fpls.2022.926277)
Supplement: Supplementary file 1 [file Data_Sheet_1.ZIP › Supplementary_materials/Supplementary methods figures and references.pdf]

Supplementary Material

Supplementary Methods

Supplementary Methods 1. Additional methodological details on improved protocol for barley culm morphological traits.

Samples were collected at Zadoks stage 90 from the second internode of barley main culm, which is considered to different extent a critical area for lodging resistance (Pinthus 1974, Berry et al., 2004). Furthermore samples at Zadoks stage 90 have a different and more uniform cellular composition compared with other growth stages (Wang et al., 2018). The second basal internode was identified as the internode following the first internode longer than 1 cm above the root crown (Berry et al., 2004; Berry et al., 2007; Berry, 2013).

At Zadock stage 90 (fully mature), three randomly selected plants for each plot were uprooted, avoiding those on the plot's borders. For each plant, the main stem was identified and the second basal internode was excised. Using a custom-made circular saw, internodes were cut in the central position to produce 5 mm thick cross-sections, taking care to produce blunt cuts. The resulting internode sections were attached with cyanoacrylic glue (Super Attak) to a black A4 cardboard, previously divided into 3cm x 5cm cells, each corresponding to a filed plot. Three samples (each from a distinct plant from the same plot) were glued in the same cell. On the side of each cardboard a paper ruler was attached in order to allow the software calibration during image analysis. Each section was then colored with a white marker (Uni-ball Posca, 0,7 mm) to ensure maximum contrast with the black background. In order to extract accurate measurements from culm sections, we developed a high-throughput image analysis protocol based on images obtained by scanning cardboards with a flat office scanner (600 dpi images in .tiff format).

The images were then analyzed to derive culm diameter and thickness data with a custom made macro command in Java language on the software ImageJ (Schindelin et al., 2012).

Supplementary Methods 2. Additional methodological details on missing genotype imputation.

To increase detection power and minimizing the loss of significant association, missing data were imputed using Beagle v5.0, which enables haplotypes inference and imputation of missing genotypes (Browning et al., 2018). Beagle uses a hidden Markov model to find the most likely haplotype pair for each individual given the genotype data for that individual. To estimate genotype phase the program works iteratively using an expectation –maximization method. Out of these markers, markers in perfect Linkage disequilibrium (LD) with adjacent SNP within the window size of 100kb (LD=1) were removed. Thus, a total of 33342 (Whole panel), 26262 (two-row subset), and 27583 (six-row subset) SNPs were left for calculation of kinship matrix and subsequent GWAS analysis.

Supplementary Methods 3. Additional methodological details on statistical analysis of phenotypic data, computation of adjusted means, estimation of variance components, and heritability.

Following a two-stage approach, in each environment (stage 1) with two replicates and coordinates of column and rows, a mixed model was used. We treated the genotype (to obtain BLUEs) and replicate as fixed effects and row and columns as random effects. Depending of the trial, the residual effects were also modelled using spatial methods that accommodate local or plot to plot variation (Table 1).

After calculation of BLUEs for each trait from all seven environments, approximately 6% of data mainly from six-row panel were missing in the genotype-environment table (i.e, for three environments JHI16, JHI17, and LUKE17) . Removing accessions with missing phenotype will reduce the sample size and consequently will negatively impact the statistical inference and subsequent GWAS analysis (Rodrigues et al., 2014; Scutari et al., 2014; Dahl et al., 2016).

Therefore, prior to subsequent analysis, and due to small fraction of missing phenotypes existed in our data, we performed imputation of missing cells in a genotype-by-environment table using the Expectation Maximization Additive Main Effects and Multiplicative Interaction (EM-AMMI) algorithm (Cauch and Zobel, 1990; Gauch 1992). We run the algorithm using five steps with the R script indicated as follows (Cauch and Zobel, 1990; Paderewski and Rodrigues, 2014): At first, initial values were assigned to missing cells; secondly, the parameters of the AMMI model were estimated; third, the adjusted means were calculated according to principal components obtained from AMMI analysis; next, missing cells were filled based on adjusted means and ;finally, the steps from 2 to 5 were repeated if the Chebyshev distance between the missing value estimations in the two progressive iteration steps were greater than the assumed precision, otherwise the algorithm was stopped. We considered the results as reliable, as the relationships between the genotypes and environments for almost all traits were present. The important factor of the algorithm is to select appropriate number of principal components to be included in imputation process. We selected this number based on the minimum of the Root Mean Square Predictive Difference (RMSPD, Gauch and Zobel, 1990; Dias and Krzanowski, 2003). The appropriate number of principal components is the one with the smallest RMSPD value. The RMSPD values were calculated according to leave-one-out cross validation (LOO-CV) procedure. Briefly, a single non-missing phenotype is hidden from the dataset and EM-AMMI is employed on training data (without missing). The procedure is repeated for each observation until no empty cell remained in the dataset. The RMSPD, is then obtained based on the difference between the hidden value and the value imputed by EM-AMMI (the predictive differences). We initially performed association analysis both on imputed data and the data after removing missing cells and found that, although the results were highly similar, the analysis with imputed phenotypes, in accordance with previous studies, resulted in well-calibrated p-values due to increased sample size (Scutari et al., 2014; Dahl et al., 2016).

In stage 2, the resulting BLUEs were used for combined analysis using a mixed model to estimate variance components, broad-sense heritability, and subsequent GWAS. Variance components and heritability values were estimated under the general form of mixed model:

$$\boldsymbol{y} = \mathrm{X}\beta + \mathrm{Z}u + \boldsymbol{\epsilon}$$

where y is a vector of observations (phenotypic BLUEs across environments), X is the design matrix for fixed effects β (intercepts and environment), Z is the design matrix for random effects (genotypes). u is the vector of random effects with $u \sim N(o.\boldsymbol{\Sigma_G})$ and $\boldsymbol{\epsilon} \sim N(o.\boldsymbol{R})$. The $\boldsymbol{\Sigma_G}$ is between environment variance-covariance matrix and **R** is a diagonal block matrix where :

$$\boldsymbol{\Sigma_G} = \begin{bmatrix} \sigma_1^2 & \cdots & \sigma_{17} \\ \vdots & \ddots & \vdots \\ \sigma_{71} & \cdots & \sigma_7^2 \end{bmatrix} \text{ and } \boldsymbol{R} = \oplus_{i=1}^{7} \boldsymbol{R}_i$$

The specification of variance structure is important in combined analysis. Traditionally the genotypic variances within all environments and the covariances between genotypic values for each pair of environments are assumes equal. We relaxed these assumptions for $\boldsymbol{\Sigma_G}$ using the mixed model allowing for unequal genotype variances and unique covariances for each pair of environments. Therefore we specified the unstructured covariance and heterogeneous variance (US) model in the multi-environment analysis (7 within-environment variances and 14 between-environment covariances). The genotype means from combined multi-environment analysis (BLUPs) were then obtained for comparisons with single environments and for correlation analysis between traits.

Using average covariance between genotypes across environments as the numerator and average variance of genotype means across environments as the denominator we estimated the heritability in the more complex case of heterogeneous genetic and error variances using the following formula (Nyquist and Baker, 1991; Holland et al., 2003; Isik et al., 2017):

$$h^2{}_g = \frac{\overline{\sigma_{gii'}}}{\frac{\overline{\sigma_{gi}^2}}{e} + \frac{(e-1)\overline{\sigma_{gii'}}}{e} + \frac{1}{e^2}\sum_{i=1}^{e}\frac{\sigma_{\epsilon i}^2}{r}}$$

where $\boldsymbol{e}$ refers to the number of environments, $\boldsymbol{r}$ refers to the number of replications within environment, $\sigma g_{ii'}$ is the genotype covariance between environments i and i', σ_{gi}^2 is the genotype variance within environment i, and $\sigma_{\epsilon i}^2$ is assumed to follow $\epsilon_i \sim N(o.\boldsymbol{R})$ in environment i and $\boldsymbol{R}$ is diagonal matrix calculated from squared errors of genotype BLUEs from stage 1. If the covariance between environments is higher, the heritability would be high accordingly. The variance parameters were estimated by maximizing the REML (Patterson and Thompson, 1971) log-likelihood function using the AI algorithm (Gilmour et al., 1995), implemented in the package ASReml-R (Butler et al., 2017). Pairwise correlations between traits based on genotype means estimated from each environment and across environments were calculated using R package ggcorrplot.

Supplementary Methods 4. Additional methodological details on multi-environment GWAS analysis.

The MTMM can be written as follow:

$$\boldsymbol{y} = \sum_{i=1}^{7} \boldsymbol{s}_i\mu_i + \mathbf{x}\beta + (\mathbf{x} \times \mathbf{l})\alpha_1 + (\mathbf{x} \times \mathbf{f})\alpha_2 + \boldsymbol{\nu}$$

Where $\boldsymbol{y}$ is the vector of phenotypic BLUEs across environments, $\mathbf{x}$ is the vector of marker scores and $\boldsymbol{s}_i$ is a vector having 1 for values belonging to the i'th environment and 0 otherwise. **l** is a vector with 1 for all the values measured in the same location, $\mathbf{f}$ is a vector with 1 for all the values measured in the same year, and $\boldsymbol{\nu} \sim N(o.\boldsymbol{\Sigma_G} \otimes \boldsymbol{K} + \boldsymbol{R})$ is a random variable comprising of both residual and random genetic effects. The variance of $\boldsymbol{\nu}$ is estimated from a variance decomposition model described above. A generalized least square (GLS) F-test was used to estimate the genome-wide marker effects depending on what kind of QTL/SNP we were interested as follows:

QF, This is the full model which tested against null model β=α_1=α_2=0 which identifies SNPs with both stable and interaction effects;

QM, To identify the main QTL which tests the model with α_1=α_2=0 against the null model with β=α_1=α_2=0;

QL, To identify the QTL × location interaction which tests the full model against the null model with α_1=0.

QY: To identify the QTL × year interaction the full model tested against null model with α_2=0.

QE: To identify any QTL × environment interaction effect where the full model is tested against null model with α_1=α_2=0.

For marker-trait association, we didn't use the Bonferroni adjustment due to its highly conservative nature and overcorrect for SNPs falling in high linkage disequilibrium that are not truly independent. Therefore, we approximated GWAS p-value significance thresholds according to the true number of 'independent SNP tests'. This effective number of SNPs was estimated in software Haploview 4.2 (Barret et al., 2005) using r-square tag threshold estimated from LD decay analysis (see LD section) (Mackay, 1996). We also retained the associations with $-\log_{10} P \geq 4$ but lower than the significance threshold as suggestive QTLs. Haploview was also used to determine the extent of QTL intervals within the barley chromosomes where SNPs detected in the same haplotype blocks were considered as the same QTL. The corresponding positions of SNPs/QTLs were then visualized using synthetic view of genomic positions of QTLs along with the circular heatmap (Pendergrass et al., 2010; Yu et al., 2018). To estimate the proportion of phenotypic variance explained by an SNP, we were faced with either a single SNP or multiple SNPs in the region with high LD between them. In the case of first situation we calculated variance using the following formulae: PVE_β (%) = $2p_i(1-p_i)\beta \times 100$ where β is the main effect derived from the GWAS model and p_i is the frequency of minor allele at SNP_i. In the case of QTL region with multiple associated SNPs, the phenotypic variance explained by the QTL was calculated as:

PVE_β (%) = ${\beta^*}^T \boldsymbol{D}^{-1} \beta^* \times 100$, where β^* is a matrix with the elements $\beta_i^* = 2p_i(1-p_i)\beta_i$ and ${\beta^*}^T$ is the transposed matrix. **D** Is the LD-matrix (Pearson correlations) of the variants in the QTL region. To derive PVE (%) explained by QTL-by-Location and QTL-by-Year effects, the $\beta_i$was replaced by α_1 and α_2 , respectively. Finally, the total phenotype variance was obtained by summing over main and interaction effects.

Supplementary Figures

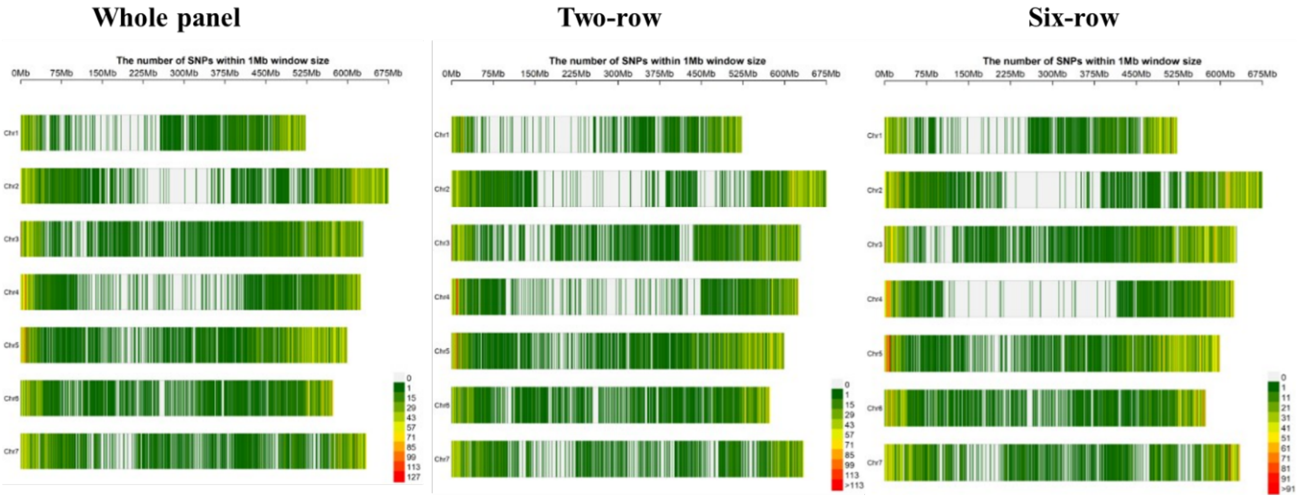

Supplementary Figure 1. Distribution of SNP markers and marker density within the window size of 1Mb within the whole panel, two-row, and six-row panels, respectively. The number of markers per each chromosome are shown in the Supplementary Table 4.

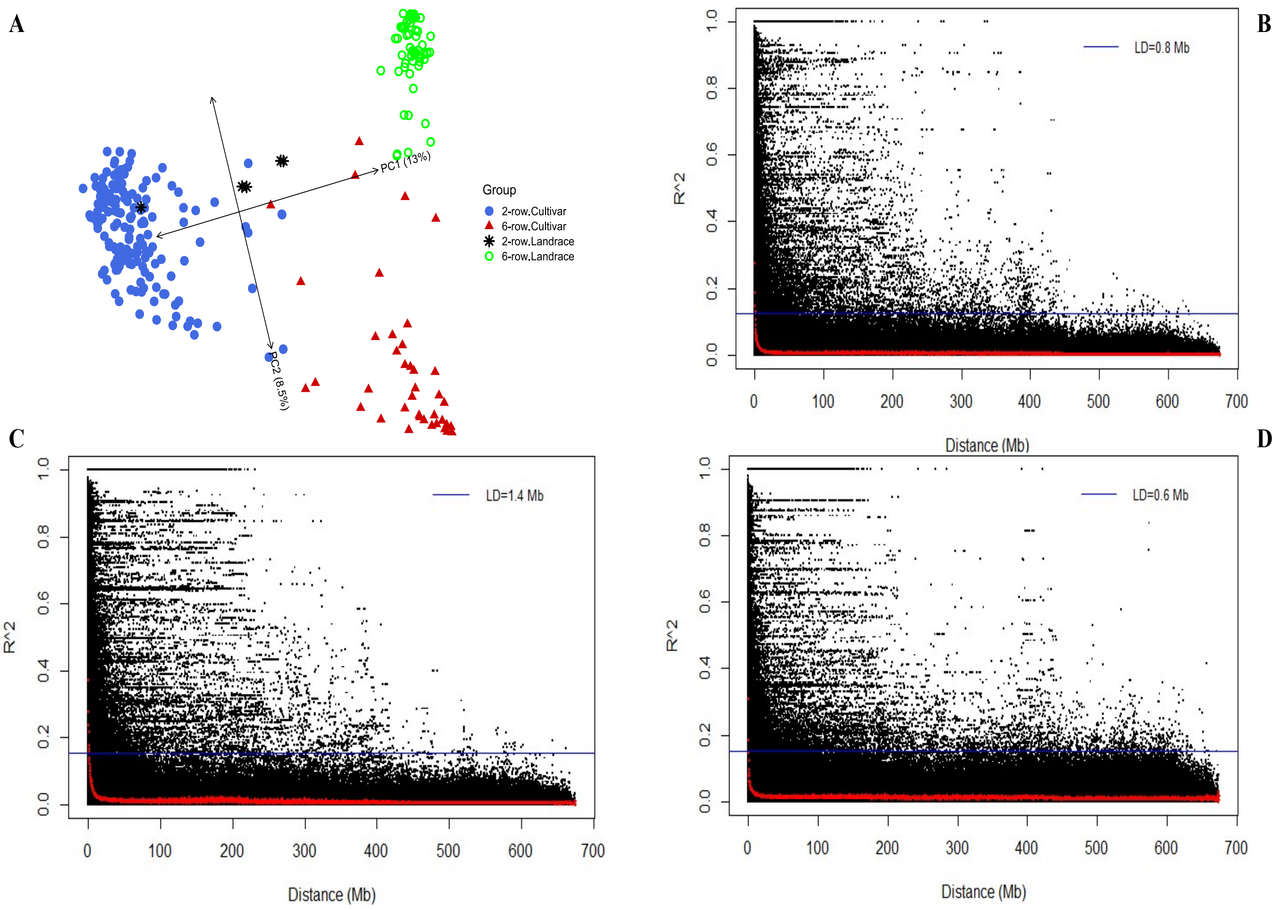

Supplementary Figure 2. A) Biplot of first two PC scores from PCA analysis conducted on genotype marker data representing population structure of the panel related to row type and germplasm resource. Plots of LD (r^2) decay corrected for population structure and relatedness representing intrachromosomal decay of marker pairs over all chromosomes as a function of physical distance. The blue line is the 95th percentile distribution of unlinked r^2 values > 50 Mb and the red line illustrates the LD decay based on LOESS fitting curve. B) Whole panel; C) two-row panel; D) six-row panel.

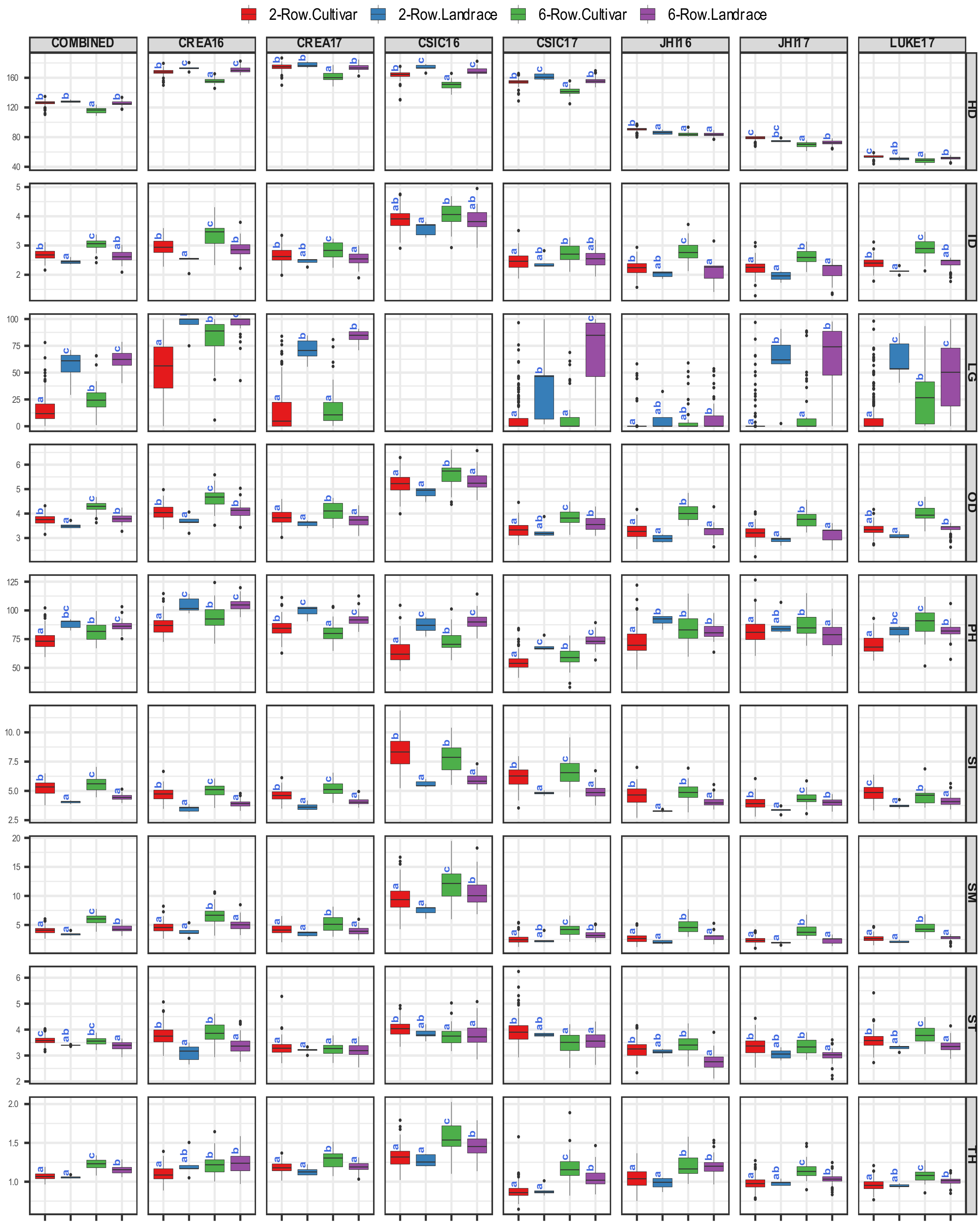

Supplementary Figure 3. Distribution of phenotypic values within row type and germplasm source based on single- and across-environment trials within the barley panel. Different letters indicate significant differences (p-value= 0.05). HD, Heading date; PH, Plant height; OD, Outer diameter; ID, Inner diameter; TH, Thickness; SM, Section modulus; ST, Stiffness; SI, Stem index; LG, Lodging.

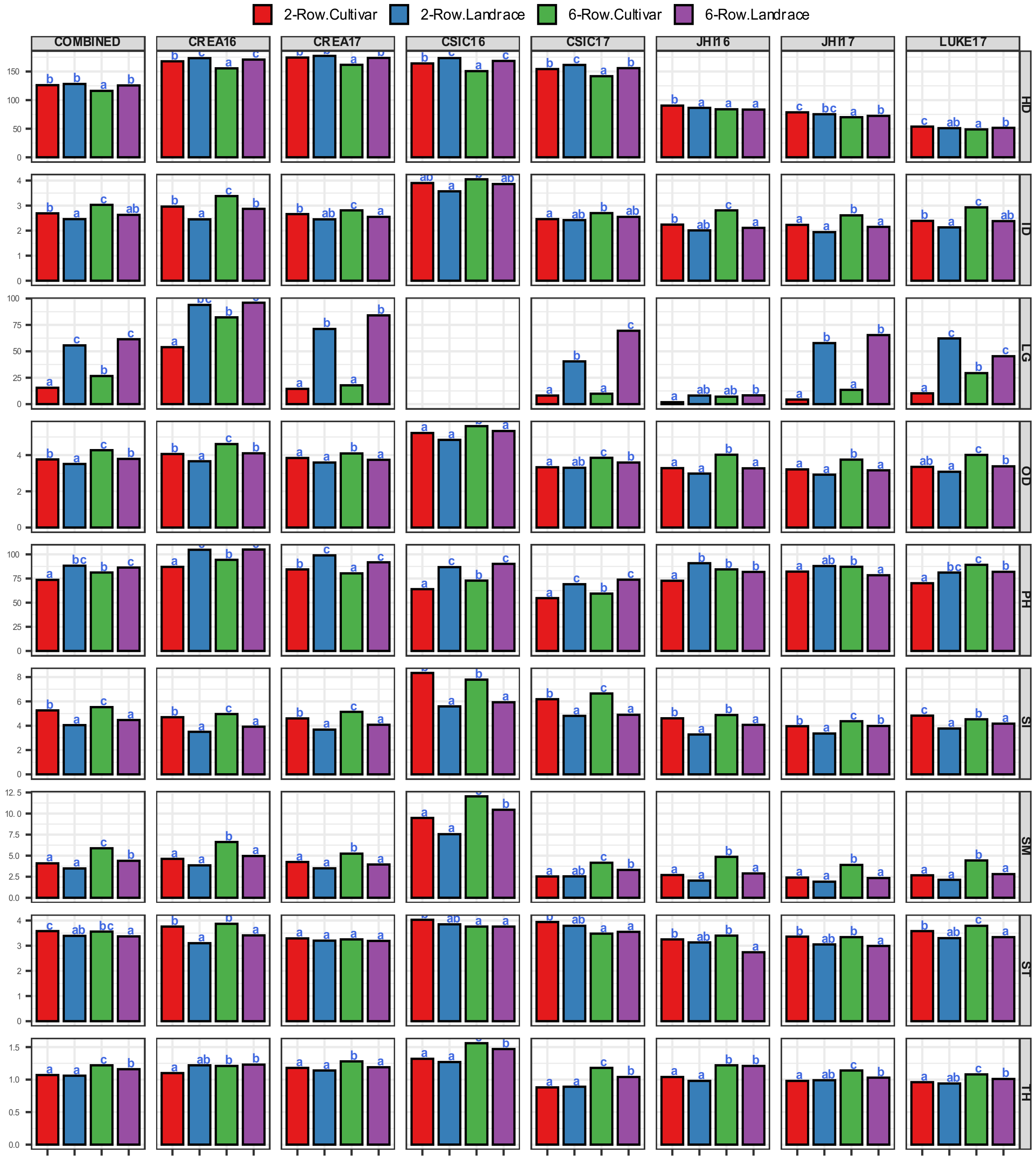

Supplementary Figure 4. Comparison of row type and germplasm source for their effect on studied traits based on single- and across-environment trials within the barley panel. Mean differences were performed using a one-way ANOVA with Tukey's honestly (HSD) test. Different letters above each column indicate significant differences (p-value= 0.05). HD, Heading date; PH, Plant height; OD, Outer diameter; ID, Inner diameter; TH, Thickness; SM, Section modulus; ST, Stiffness; SI, Stem index; LG, Lodging.

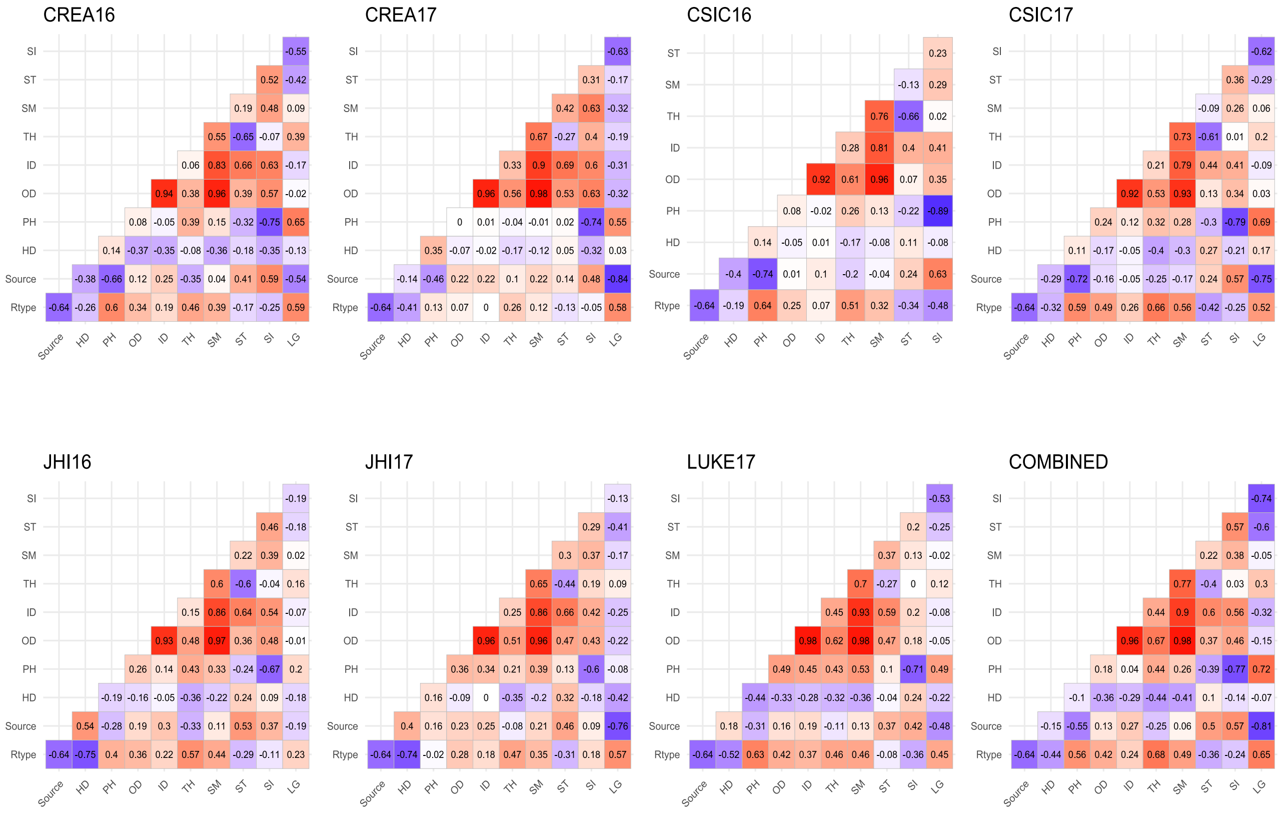

Supplementary Figure 5. Pairwise correlation coefficients between traits, row type, and germplasm source (cultivar/landrace) in the whole panel based on genotype values estimated both in single and combined multi-environment analysis. Data for lodging In CSIC16 is not available. HD, Heading date; PH, Plant height; OD, Outer diameter; ID, Inner diameter; TH, Thickness; SM, Section modulus; ST, Stiffness; SI, Stem index; LG, Lodging.

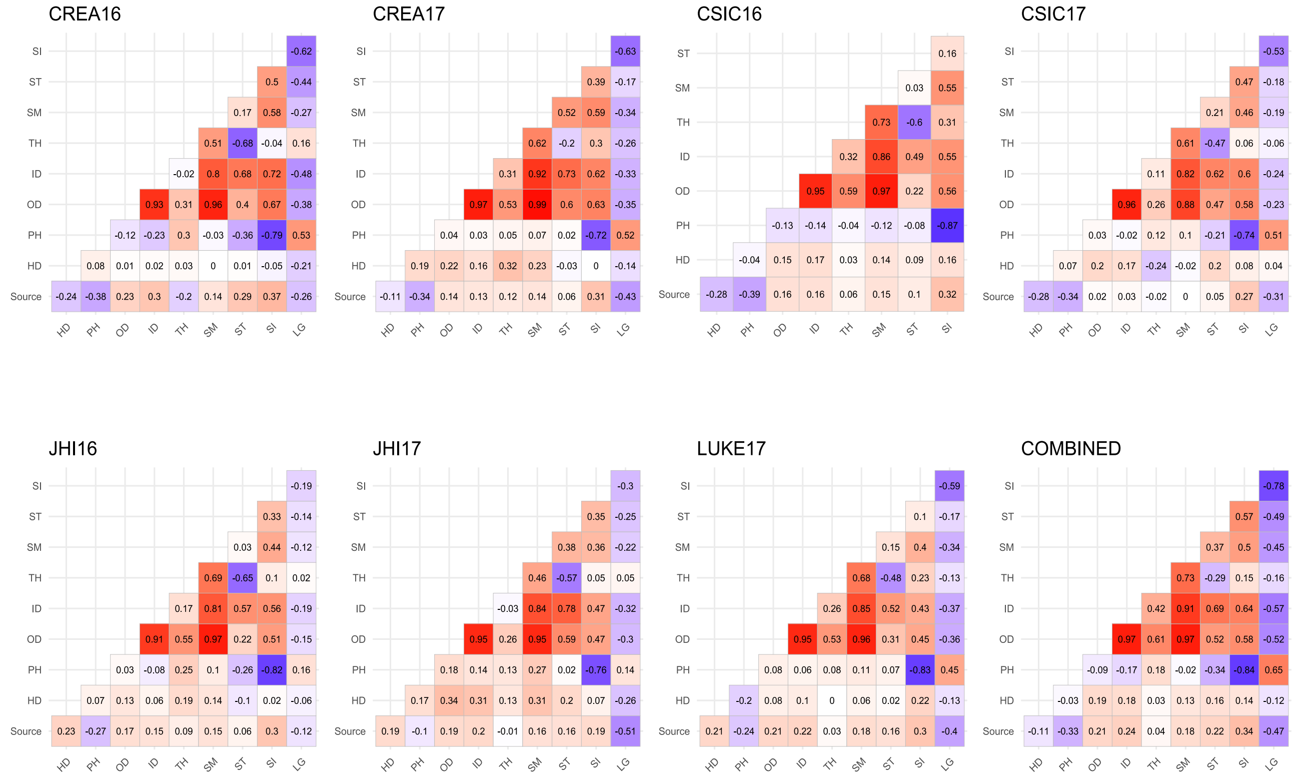

Supplementary Figure 6. Pairwise correlation coefficients between traits and germplasm source (cultivar/landrace) in the two-row panel based on genotype values estimated both in single and combined multi-environment analysis. Data for lodging In CSIC16 is not available. HD, Heading date; PH, Plant height; OD, Outer diameter; ID, Inner diameter; TH, Thickness; SM, Section modulus; ST, Stiffness; SI, Stem index; LG, Lodging.

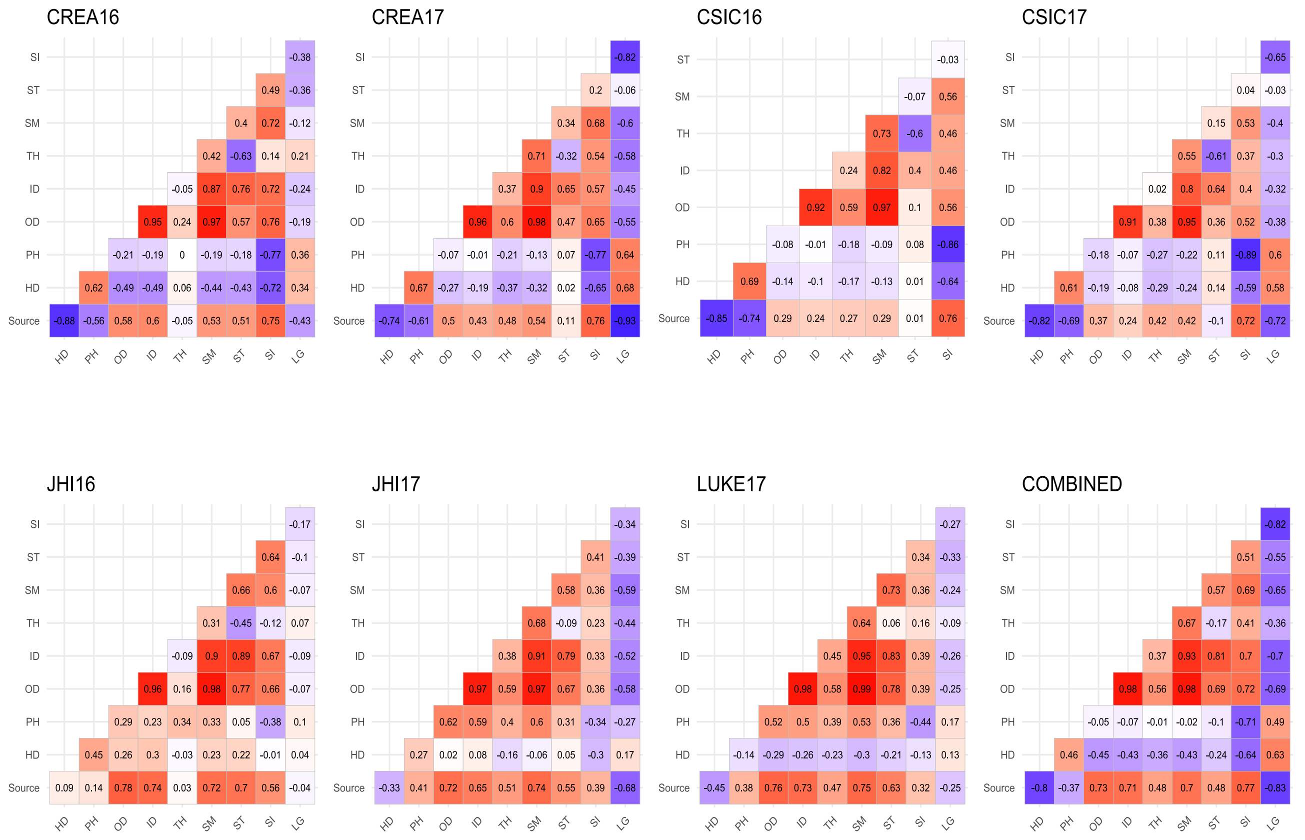

Supplementary Figure 7. Pairwise correlation coefficients between traits and germplasm source (cultivar/landrace) in the six-row panel based on genotype values estimated both in single and combined multi-environment analysis. Data for lodging In CSIC16 is not available. HD, Heading date; PH, Plant height; OD, Outer diameter; ID, Inner diameter; TH, Thickness; SM, Section modulus; ST, Stiffness; SI, Stem index; LG, Lodging.

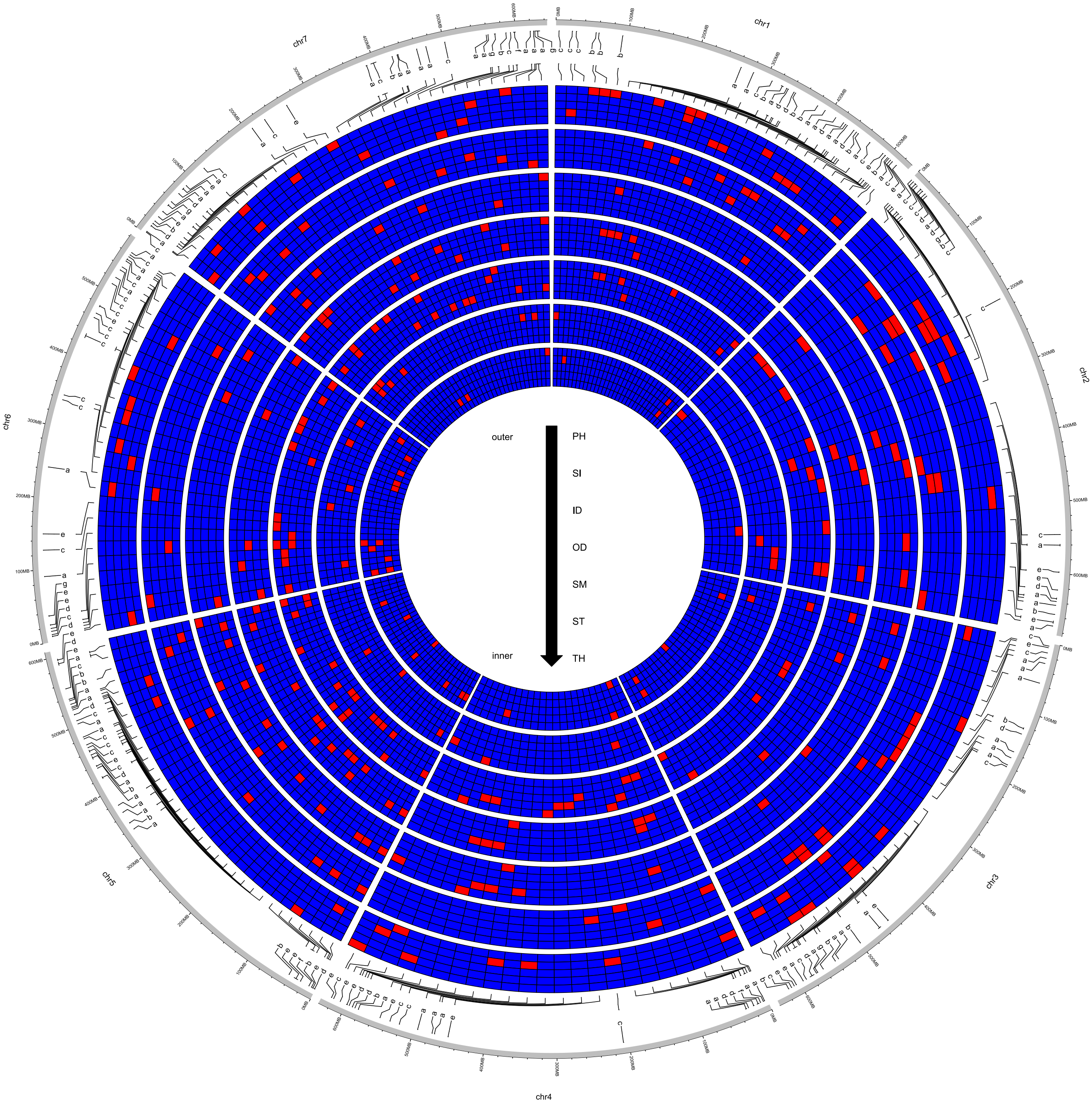

Supplementary Figure 8. Circos heatmap for the 192 QTLs identified from GWAS of seven traits for the whole panel and row type groups. Each track belongs to one trait which also divided into five subsectors for QF, QM, QE, QL, and QY effect with red colors showing the presence of QTL at that position. The letters a, b, c, d, e, and f are respectively related to QTLs identified in whole panel (a), two-row (b), six-row (d), both whole panel and two-row (e), both whole panel and six-row (f), and both two-row and six-row (g), and all the panels (h). PH, Plant height; OD, Outer diameter; ID, Inner diameter; TH, Thickness; SM, Section modulus; ST, Stiffness; SI, Stem index.

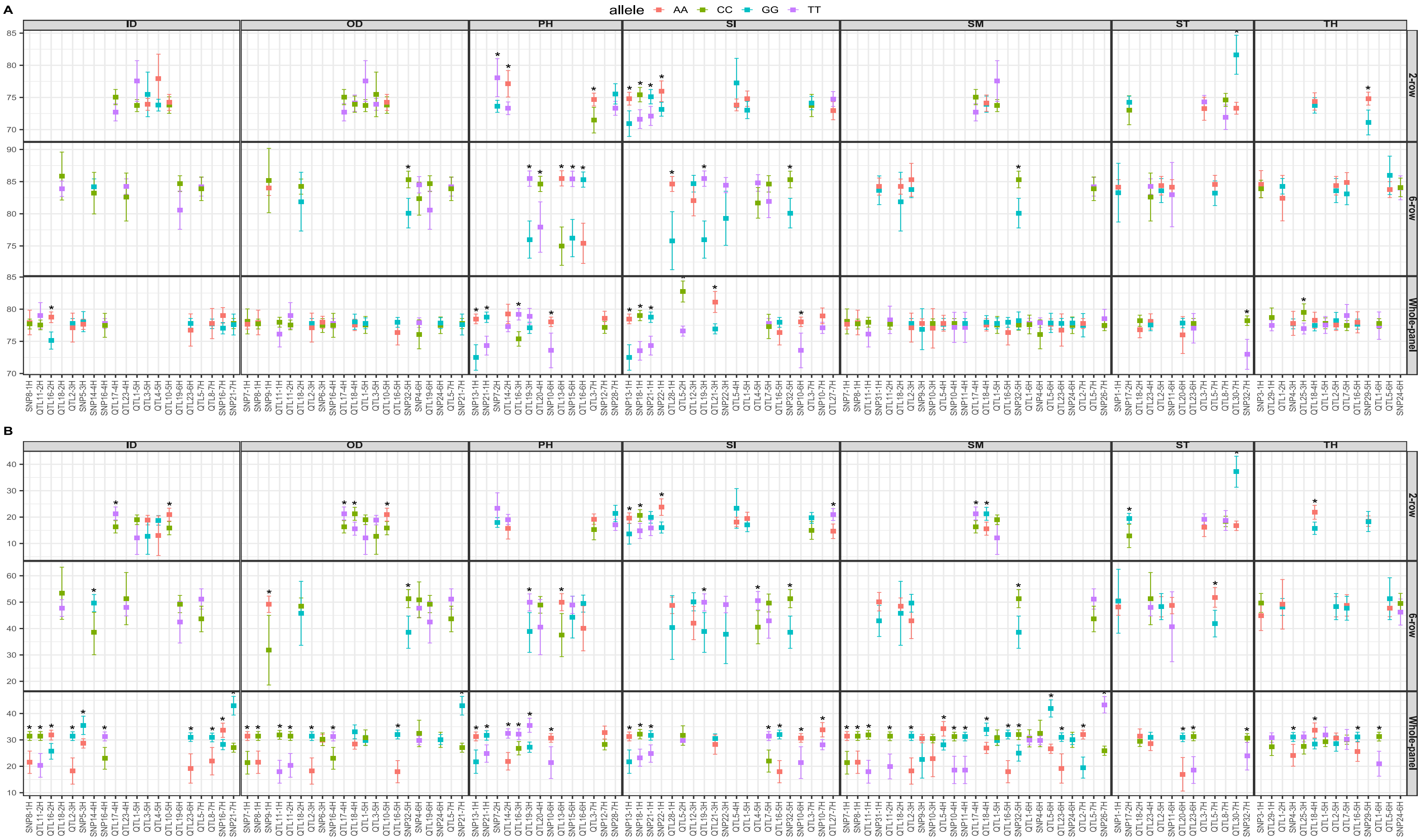

Supplementary Figure 9. Comparison of allelic variants at peak markers of loci with QM effect (Supplementary Table 7). **A**) comparison between alleles at each marker for their effect on plant height; **B**) and their effect on lodging. The points indicate the mean value and the bars indicate the 95% confidence interval of the mean of corresponding allele. Significant differences are shown with asterisk. PH, Plant height; OD, Outer diameter; ID, Inner diameter; TH, Thickness; SM, Section modulus; ST, Stiffness; SI, Stem index.

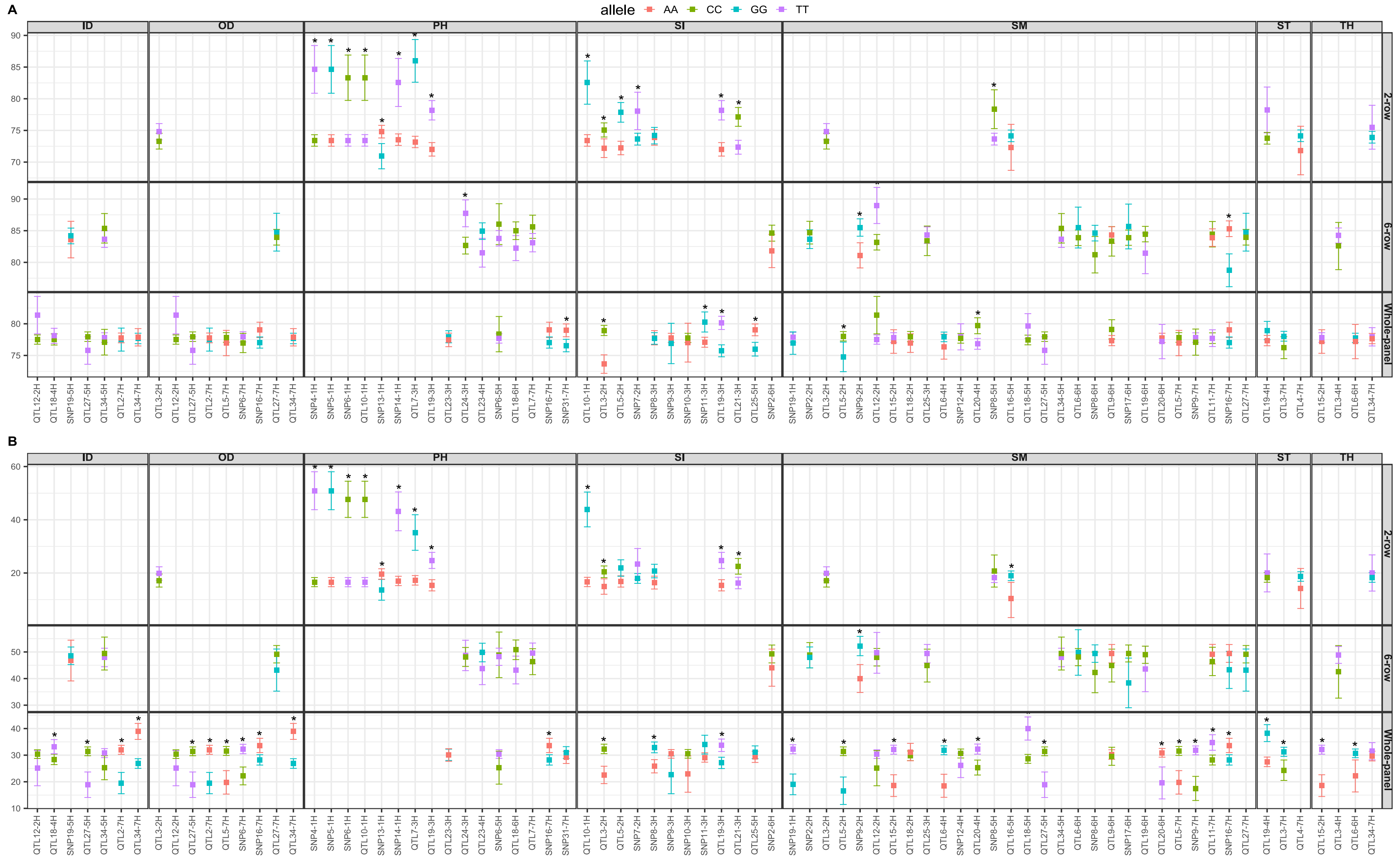

Supplementary Figure 10. Comparison of allelic variants at peak markers of loci with QF effect (Supplementary Table 7). **A**) comparison between alleles at each marker for their effect on plant height; **B**) and their effect on lodging. The points indicate the mean value and the bars indicate the 95% confidence interval of the mean of corresponding allele. Significant differences are shown with asterisk. PH, Plant height; OD, Outer diameter; ID, Inner diameter; TH, Thickness; SM, Section modulus; ST, Stiffness; SI, Stem index; LG, Lodging.

REFERENCES

Barrett, J. C., Fry, B., Maller, J., and Daly, M. J. (2005). Haploview: analysis and visualization of LD and haplotype maps. Bioinformatics. 21, 263–265. doi: 10.1093/bioinformatics/bth457

Berry, P. M. (2013). "Lodging resistance in cereals in sustainable food production," in Sustainable Food Production, eds P. Christou, R. Savin, B. A. Costa-Pierce, I. Misztal, and C. B. A. Whitelaw (Berlin: Springer), 1096–1110.

Berry, P. M., Sterling, M., Spink, J. H., Baker, C. J., Sylvester-Bradley, R., Mooney, S. J., et al. (2004). "Understanding and reducing lodging in cereals," in Advances in Agronomy, ed D. L. Sparks (Cambridge, MA: Academic Press), 217–271. doi: 10.1016/S0065-2113(04)84005-7

Berry, P. M., Sylvester-Bradley, R., and Berry, S. (2007). Ideotype design for lodging-resistant wheat. Euphytica. 154, 165–179. doi: 10.1007/s10681-006-9284-3

Browning, B. L., Zhou, Y., and Browning, S. R. (2018). A one-penny imputed genome from next-generation reference panels. Am. J. Hum. Genet. 103, 338–348. doi: 10.1016/j.ajhg.2018.07.015.

Butler, D. G., Cullis, B. R., Gilmour, A. R., Gogel, B. J., and Thompson, R. (2017). ASReml-R Reference Manual Version 4. Hemel Hempstead, UK: VSN International Ltd.

Gauch, H. G., and Zobel, R. W. (1990). Imputing missing yield trial data. Theor. Appl. Genet. 79, 753–761. doi: 10.1007/bf00224240

Gauch, H. G. (1992). Statistical Analysis of Regional Yield Trials: AMMI Analysis of Factorial Designs. Amsterdam: Elsevier.

Dahl, A., Iotchkova, V., Baud, A., Johansson, S., Gyllensten, U., Soranzo, N., et al. (2016). A multiple-phenotype imputation method for genetic studies. Nat. Genet. 48, 466–472. doi: 10.1038/ng.3513

Dias, C. T. D., and Krzanowski, W. (2003). Model selection and cross validation in additive main effect and multiplicative interaction models. Crop. Sci. 43, 865–873. doi: 10.2135/cropsci2003.8650

Gilmour, A. R., Thompson, R., and Cullis, B. R. (1995). Average information REML: an efficient algorithm for variance parameter estimation in linear mixed models. Biometrics. 51, 1440–1450. doi: 10.2307/2533274

Holland, J. (2003). Estimating and interpreting heritability for plant breeding: an update. Plant Breed. Rev. 22:9–112. doi: 10.1002/9780470650202.ch2

Isik, F., Holland, J., Maltecca, C. (2017). Genetic Data Analysis for Plant and Animal Breeding (Cham: Springer International Publishing). doi: 10.1007/978-3-319-55177-7

Mackay, T. F. C. (1996). The nature of quantitative genetic variation revisited: Lessons from Drosophila bristles. BioEssays. 18: 113–121. doi: 10.1002/bies.950180207

Nyquist, W. E., Baker, R. (1991). Estimation of heritability and prediction of selection response in plant populations. Crit. Rev. Plant Sci. 10, 235–322. doi: 10.1080/07352689109382313

Paderewski, J., and Rodrigues, P. C. (2014). The usefulness of EM-AMMI to study the influence of missing data pattern and application to Polish post-registration winter wheat data. Austral. J. Crop. Sci. 8, 640–645.

Patterson, H. D., and Thompson, R. (1971). Recovery of interblock information when block sizes are unequal. Biometrika. 58, 545–554. doi: 10.1093/biomet/58.3.545

Pendergrass, S., Dudek, S., Crawford, D., and Ritchie, M. (2010). Synthesis-view: visualization and interpretation of SNP association results for multi-cohort, multi-phenotype data and meta-analysis. BioData. Min. 3:10. doi: 10.1186/1756-0381-3-10

Pinthus, M. J. (1974). Lodging in wheat, barley, and oats: the phenomenon, its causes, and preventive measures. Adv. Agron. 25, 209–263. doi: 10.1016/s0065-2113(08)60782-8

Rodrigues, P. C., Malosetti, M., Gauch, H. G., and Van Eeuwijk, F. A. (2014). A weighted AMMI algorithm to study genotype- by-environment interaction and QTL-by-environment interaction. Crop. Sci. 54, 1555–1570.

Schindelin, J., Arganda-Carreras, I., Frise, E., Kaynig, V., Longair, M., Pietzsch, T., et al. (2012). Fiji: an open-source platform for biological-image analysis. Nat. Methods. 9, 676–682. doi: 10.1038/nmeth.2019

Scutari, M., Howell, P., Balding, D. J., and Mackay, I. (2014). Multiple quantitative trait analysis using bayesian networks. Genetics 198, 129–137. doi: 10.1534/genetics.114.165704

Wang, B., Smith, S. M., and Li, J. (2018). Genetic regulation of shoot architecture. Annu. Rev. Plant Biol. 69, 437–468. doi: 10.1146/annurev-arplant-042817-040422

Yu, Y., Ouyang, Y., and Yao, W. (2018). shinyCircos: an R/Shiny application for interactive creation of Circos plot. Bioinformatics 34, 1229–1231. doi: 10.1093/bioinformatics/btx763
